# Beginner’s guide to comparative bacterial genome analysis using next-generation sequence data

**DOI:** 10.1186/2042-5783-3-2

**Published:** 2013-04-10

**Authors:** David J Edwards, Kathryn E Holt

**Affiliations:** 1 Department of Biochemistry and Molecular Biology, Bio21 Institute, University of Melbourne, Victoria 3010, Australia; 2 Victorian Life Sciences Computation Initiative, University of Melbourne, Victoria, 3010, Australia

**Keywords:** Bacterial, Microbial, Comparative, Genomics, Next generation sequencing, Analysis, Methods

## Abstract

High throughput sequencing is now fast and cheap enough to be considered part of the toolbox for investigating bacteria, and there are thousands of bacterial genome sequences available for comparison in the public domain. Bacterial genome analysis is increasingly being performed by diverse groups in research, clinical and public health labs alike, who are interested in a wide array of topics related to bacterial genetics and evolution. Examples include outbreak analysis and the study of pathogenicity and antimicrobial resistance. In this beginner’s guide, we aim to provide an entry point for individuals with a biology background who want to perform their own bioinformatics analysis of bacterial genome data, to enable them to answer their own research questions. We assume readers will be familiar with genetics and the basic nature of sequence data, but do not assume any computer programming skills. The main topics covered are assembly, ordering of contigs, annotation, genome comparison and extracting common typing information. Each section includes worked examples using publicly available *E. coli* data and free software tools, all which can be performed on a desktop computer.

## Review

### Introduction and aims

High throughput sequencing is now fast and cheap enough to be considered part of the toolbox for investigating bacteria [1,2]. This work is performed by diverse groups of individuals including researchers, public health practitioners and clinicians, interested in a wide array of topics related to bacterial genetics and evolution. Examples include the study of clinical isolates as well as laboratory strains and mutants [3]; outbreak investigation [4,5]; and the evolution and spread of drug resistance [6]. Bacterial genome sequences can now be generated in-house in many labs, in a matter of hours or days using benchtop sequencers such as the Illumina MiSeq, Ion Torrent PGM or Roche 454 FLX Junior [1,2]. Much of this data is available in the public domain, allowing for extensive comparative analysis; *e.g.* in February 2013 the GenBank database included >6,500 bacterial genome assemblies, two thirds of which were in ‘draft’ form (*i.e.* presented as a set of sequence fragments rather than a single sequence representing the whole genome, see [7] for a detailed discussion).

In this beginner’s guide, we aim to provide an entry point for individuals wanting to make use of whole-genome sequence data for the *de novo* assembly of genomes to answer questions in the context of their broader research goals. The guide is not aimed at those wishing to perform automated processing of hundreds of genomes at a time; some discussion of the use of sequencing in routine microbiological diagnostic laboratories is available in the literature [8]. We assume readers will be familiar with genetics and the basic nature of sequence data, but do not assume any computer programming skills and all the examples we use can be performed on a desktop computer (Mac, Windows or Linux). The guide is not intended to be exhaustive, but to introduce a set of simple but flexible and free tools that can be used to investigate a variety of common questions including (i) how does this genome compare to that one?, and (ii) does this genome have plasmids, phage or resistance genes? Each section includes guidance on where to find more detailed technical information, alternative software packages and where to look for more sophisticated approaches.

### Examples and tutorial

Throughout the guide, we will use *Escherichia coli* O104:H4 as a worked example. *E. coli* O104:H4 was responsible for a lethal foodborne outbreak of haemolytic uraemic syndrome (HUS) in Germany during 2011 [9-11]. Sequence reads and assemblies from a number of outbreak strains, generated using different high throughput sequencing platforms (including Illumina, Ion Torrent and 454) are now available for download from the European Nucleotide Archive [11-17].

The outbreak strain belongs to an enteroaggregative *E. coli* (EAEC) lineage that has acquired a bacteriophage encoding Shiga-toxin (commonly associated with enterohaemorrhagic *E. coli* (EHEC)), and multiple antibiotic resistance genes [12]. For the worked examples, we will use a set of paired-end Illumina reads from O104:H4 strain TY-2482 (ENA accession SRR292770), but also include alternatives for the other available short-read data types. For those so interested, longer Pacific Bioscience reads are also available, but are not included in this tutorial.

The workflow has been divided into five logical sections: assembly, ordering of contigs, annotation, genome comparison and typing. Examples using *E. coli* O104:H4 data are presented in the text and figures, and detailed instructions on how to replicate the example are provided in the corresponding tutorial (Additional file 1). The tutorial includes links to the software programs required for each stage, the specific steps needed to use the program(s), and the expected inputs and outputs (instructions for software installation are provided by the developers of each program).

Whilst quality control of raw sequence data can be important in obtaining the best assembly for comparison, the number and complexity of possible steps is too numerous, and the variations between platforms too substantial, to cover in this guide. However, we recommend readers check the quality of raw sequence reads using the tools accompanying their benchtop sequencing machines, or use FastQC to assess the quality of raw read sets (see Tutorial, Additional file 1).

### Genome assembly

**Figure 1:**
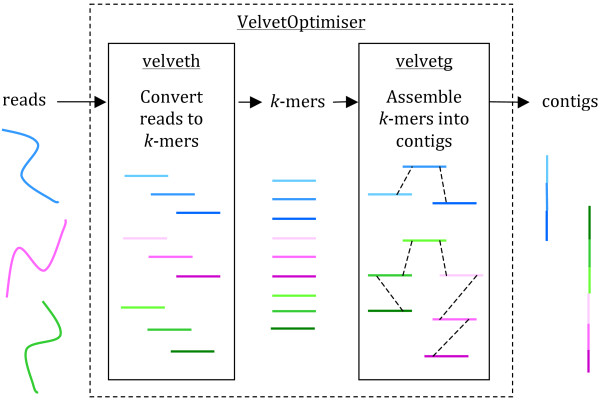
**Genome assembly with Velvet. **Reads are assembled into contigs using *Velvet* and *VelvetOptimiser *in two steps, (1) *velveth *converts reads to *k*-mers using a hash table, and (2) *velvetg *assembles overlapping *k*-mers into contigs via a de Bruijn graph. *VelvetOptimiser *can be used to automate the optimisation of parameters for *velveth *and *velvetg *and generate an optimal assembly. To generate an assembly of *E. coli *O104:H4 using the command-line tool *Velvet*: • Download *Velvet *[23] (we used version 1.2.08 on Mac OS X, compiled with a maximum *k*-mer length of 101 bp) • Download the paired-end Illumina reads for *E. coli* O104:H4 strain TY-2482 (ENA accession SRR292770 [17]) • Convert the reads to *k*-mers using this command: velveth out_data_35 35 -fastq.gz -shortPaired -separate SRR292770_1.fastq.gz SRR292770_2.fastq.gz • Then, assemble overlapping *k*-mers into contigs using this command: velvetg out_data_35 -clean yes -exp_cov 21 -cov_cutoff 2.81 -min_contig_lgth 200 This will produce a set of contigs in multifasta format for further analysis. See Additional file 1: Tutorial for further details, including help with downloading reads and using *VelvetOptimiser*.

*De novo* assembly is the process of merging overlapping sequence reads into contiguous sequences (contigs) without the use of any reference genome as a guide (Figure 1). The most efficient assemblers for short-read sequences are typically those that employ de Bruijn graphs to produce an assembly [18]. An eloquent explanation of how de Bruijn graphs work in sequence assembly can be found in Compeau *et al.*[18]. One of the first and most widely used de Bruijn graph assemblers is the open-source program *Velvet*[19]. With further development to improve the resolution of repeats and scaffolding using paired-end and longer reads [20], *Velvet* remains one of the most-used (and cited) assemblers for bacterial genomes, being best suited to Illumina sequence reads (*Velvet* is included as the default assembler in the Illumina MiSeq analysis suite).

Ion Torrent reads are better assembled using the open source program *MIRA*[21], which uses a modified Smith-Waterman algorithm for local alignment rather than a de Bruijn graph method. *MIRA* is available as a plugin for the Ion Torrent analysis suite. For 454 data, Roche provides a proprietary (de Bruijn graph-based) assembler [22].

When using a de Bruijn graph assembler, a number of variables need to be considered in order to produce optimal contigs [23]. This can be automated quite effectively using *VelvetOptimiser*[24]. The key issue is selecting an appropriate *k*-mer length for building the de Bruijn graph. Different sequencing platforms produce fragments of differing length and quality [1], meaning very different ranges of *k*-mers will be better suited to different types of read sets. A balance must be found between the sensitivity offered by a smaller *k*-mer against the specificity of a larger one [18]. Other variables to consider when running *Velvet* include the expected coverage across the genome, the length of the insert sizes in paired-end read libraries, and the minimum coverage (read depth) cut-off value, all of which can be automated using *VelvetOptimiser*[23]. If the coverage obtained is higher than 20× reads deep on average, the chances of errors being incorporated into the contigs increases, as de Bruijn graph assemblers cannot distinguish between an error and a real variant if there is lots of evidence for the error, as found with higher coverage levels. In this case, a subset of the reads can be sampled and used for the assembly [23].

Instructions on how to assemble Illumina reads from *E. coli* O104:H4 strain TY-2482 using *Velvet* are given in Figure 1 and Additional file 1: Tutorial. The assembler takes the sequence reads as input (in fastq format) and outputs the assembled contigs (in multifasta format). Note that the contig set, referred to as the draft assembly, will include sequences derived from all the DNA present in the sequenced sample, including chromosome(s) and any bacteriophage or plasmids.

### Ordering and viewing assembled contigs

Once a set of contigs have been assembled from the sequencing reads, the next step is to order those contigs against a suitable reference genome. This may seems counter-intuitive at first as we have applied *de novo* assembly to obtain these contigs, but ordering the contigs aids the discovery and comparison process. The best reference to use is usually the most closely related bacterium with a ‘finished’ genome, but as in the case of *E. coli* O104:H4, finding the best reference may itself involve trial and error [12].

Ordering of contigs can be achieved using command-line tools such as *MUMmer*[25], which can be simplified using a wrapper program like *ABACAS*[26]. However we suggest the easiest way for beginners is to use the contig ordering tool in the Java-based graphical-interface program *Mauve*[27,28]. This ordering algorithm uses an iterative mapping approach to find the best fit for each contig against the reference genome. *Mauve* takes as input the reference genome in fasta format along with the assembly in multifasta format, and outputs another multifasta file containing the ordered contigs. Detailed instructions for ordering the *E. coli* O104:H4 contigs against a reference are given in Additional file 1: Tutorial.

Due to evolutionary differences between the reference and novel genome, the presence of (often mobile) repeat elements such as prophages, and the very nature of short-read assemblers, there will almost certainly be assembly errors present within the contigs. Indeed, all assemblers used in Assemblathon 1 [29] and the Genome Assembly Gold-standard Evaluations (GAGE) [30] community “bake-offs” produced assemblies with errors. The error rate of an assembly can be assessed if a closely related reference genome is available. A good option for assessing the error rate is *MauveAssemblyMetrics*[31] (see Additional file 1: Tutorial for an example with *E. coli* O104:H4), an optional addition to *Mauve* that generates a report on assembly quality.

**Figure 2:**
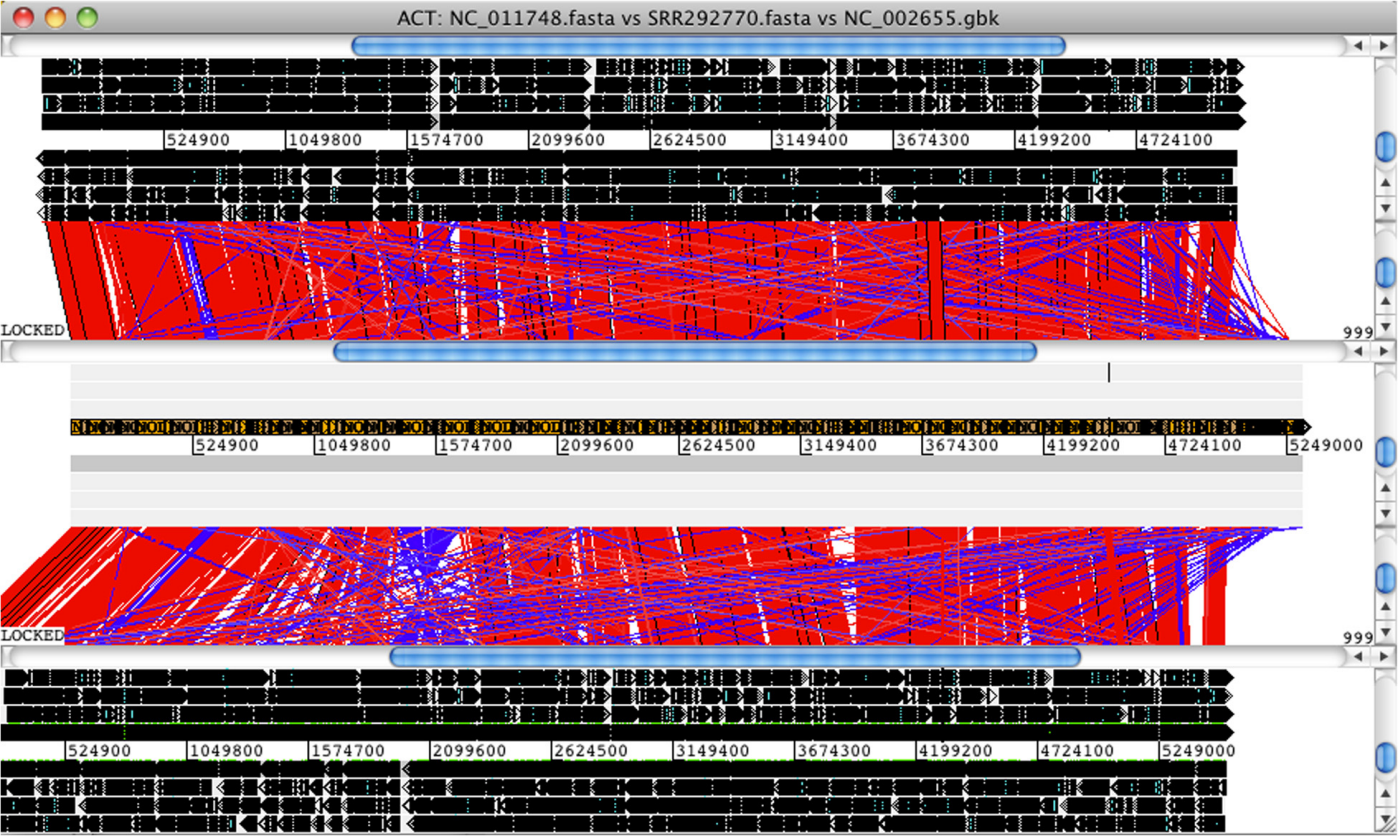
**Pairwise genome comparisons with ACT, the Artemis Comparison Tool.***Artemis *and *ACT *are free, interactive genome browsers [32,40] (we used *ACT *11.0.0 on Mac OS X). • Open the assembled *E. coli *O104:H4 contigs in *Artemis *and write out a single, concatenated sequence using File -> Write -> All Bases -> FASTA Format. • Generate a comparison file between the concatenated contigs and 2 alternative reference genomes using the website *WebACT *(http://www.webact.org/). • Launch *ACT *and load in the reference sequences, contigs and comparison files, to get a 3-way comparison like the one shown here. Here, the *E. coli *O104:H4 contigs are in the middle row, the enteroaggregative *E. coli *strain Ec55989 is on top and the enterohaemorrhagic *E. coli *strain EDL933 is below. Details of the comparison can be viewed by zooming in, to the level of genes or DNA bases.

Another way to explore the ordered assembly is by means of visualization. *Mauve* provides one way to visualize the assembly by alignment to other sequences (see Additional file 1: Tutorial for instructions). Another option is to use *Artemis* and the companion *Artemis Comparison Tool (ACT*), a pair of open-source Java-based applications [32]. An example using *E. coli* O104:H4 is shown in Figure 2 and in Additional file 1: Tutorial. To view comparisons in *ACT*, you need to first generate a comparison file that identifies regions of homology between your assembly and a reference genome. You can then load this into ACT along with your assembly and reference sequence(s). The comparison file can be generated using the *WebACT* or *DoubleACT* websites, or using *BLAST+* on your own computer (see Additional file 1: Tutorial for details of these programs). Note that before you can generate the comparison file, the assembly needs to be converted into a single fasta sequence. This can be done in *Artemis* (Figure 2)*,* or using a command-line tool such as the ‘union’ command in the *EMBOSS* package [33] (see Additional file 1: Tutorial for details).

### Genome annotation

Once the ordered set of contigs has been obtained, the next step is to annotate the draft genome. Annotation is the process of ‘gene’ finding, and can also include the identification of ribosomal and transfer RNAs encoded in the genome. Bacterial genome annotation is most easily achieved by uploading a genome assembly to an automated web-based tool such as *RAST*[34,35]. There are also many command-line annotation tools available. These include methods based on *de novo* discovery of genes, such as *Prokka*[36] and *DIYA*[37], or programs that transfer annotation directly from closely related genomes, such as *RATT*[38] and *BG-7*[39].

Since the quality of the final annotation is largely determined by the quality of the gene database used, we prefer the easy-to-use online *de novo* annotation tool *RAST* for bacterial genome annotation [35]. *RAST* takes as input the ordered contigs in multifasta format, identifies open reading frames that are likely to be genes, and uses a series of subsystem techniques (the ‘ST’ in *RAST*) to compare these with a sophisticated database of genes and RNA sequences, producing a high-quality annotation of the assembly. The genes identified can be viewed, and compared to other genomes, using the *RAST* online tool. The annotation can also be downloaded in a variety of formats, including in GenBank format. See Additional file 1: Tutorial for detailed instructions on how to annotate the *E. coli* O104:H4 genome using *RAST*.

### Comparative genome analysis

For most sequencing experiments, comparison to other genomes or sequences is a critical step. Sometimes general questions are asked, along the lines of “which genes do these genomes share and which are unique to particular genomes?”. In many cases, users are also interested in looking for specific genes that are known to have important functions, such as virulence genes or drug resistance determinants.

For most users, it is important to be able to visualize these comparisons, both to aid understanding and interpretation of the data, and to generate figures for communicating results. We therefore recommend three software tools that combine data analysis and visualization - *BRIG*, *Mauve* and *ACT* (the latter two have already been introduced above). For more experienced users, comparative questions can also be answered using command-line search tools, such as *MUMmer* or *BLAST*.

*ACT*[32,40] is a Java-based tool for visualizing pairwise comparisons of sequences, including whole genomes. As outlined above, *BLAST* is used to compare the sequences (this can be done locally, or through web services); the two genomes and the *BLAST* result are then loaded into *ACT* for visualization of the comparison (see Additional file 1: Tutorial). Multiple pairwise comparisons can be visualized simultaneously; an example using *E. coli* O104:H4 is given in Figure 2 and Additional file 1: Tutorial. Regions of sequence homology are linked by blocks, which are coloured red (same orientation) or blue (reverse orientation), with saturation indicating the degree of homology (dark=high homology, to light=low homology). Advantages of using *ACT* include (i) the flexibility to zoom right out to see whole-genome comparisons, (ii) ability to zoom right down to DNA and/or protein sequences to examine fine-scale comparisons, and (iii) it is possible to add or edit annotations for the genomes being compared.

**Figure 3:**
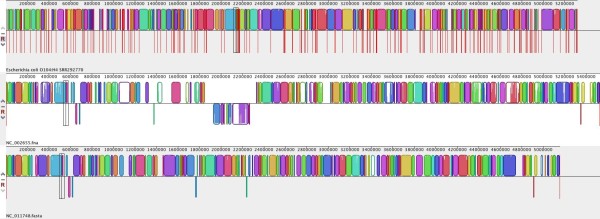
**Mauve for multiple genome alignment. **Mauve is a free alignment tool with an interactive browser for visualising results [27,41] (we used Mauve 2.3.1 on Mac OS X). • Launch Mauve and select File -> Align with progressiveMauve • Click ‘Add Sequence…’ to add your genome assembly (e.g. annotated *E. coli *O104:H4 contigs) and other reference genomes for comparison. • Specify a file for output, then click ‘Align…’ • When the alignment is finished, a visualization of the genome blocks and their homology will be displayed, as shown here. *E. coli *O104:H4 is on the top, red lines indicate contig boundaries within the assembly. Sequences outside coloured blocks do not have homologs in the other genomes.

*Mauve* is a Java-based tool for multiple alignment of whole genomes, with a built-in viewer and the option to export comparative genomic information in various forms [27,41]. Its alignment functions can also be used to order and orient contigs against an existing assembly, as outlined above. *Mauve* takes as input a set of genome assemblies, and generates a multiple whole-genome alignment. It identifies blocks of sequence homology, and assigns each block a unique colour. Each genome can then be visualized as a sequence of these coloured sequence blocks, facilitating visualization of the genome comparisons. An example is given in Figure 3. This makes it easy to identify regions that are conserved among the whole set of input genomes, and regions that are unique to subsets of genomes (islands). The tutorial (Additional file 1) includes a detailed example of how to use *Mauve* to identify unique regions in the *E. coli* O104:H4 outbreak assembly compared to EHEC and EAEC chromosomal sequences. Because *Mauve* generates an alignment of the genome sequences, it can also be used to identify single nucleotide polymorphisms (SNPs, or point mutations) suitable for downstream phylogenetic or evolutionary analyses (see the *Mauve* user guide for details).

**Figure 4:**
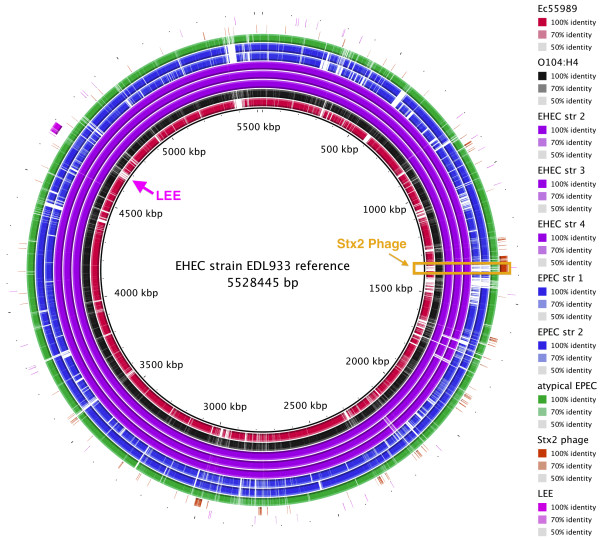
**BRIG for multiple genome comparison. **BRIG is a free tool [42,43] that requires a local installation of BLAST (we used BRIG 0.95 on Mac OS X). The output is a static image. • Launch BRIG and set the reference sequence (EHEC EDL933 chromosome) and the location of other *E. coli *sequences for comparison. If you include reference sequences for the Stx2 phage and LEE pathogenicity island, it will be easy to see where these sequences are located. • Click ‘Next’ and specify the sequence data and colour for each ring to be displayed in comparison to the reference. • Click ‘Next’ and specify a title for the centre of the image and an output file, then click ‘Submit’ to run BRIG. • BRIG will create an output file containing a circular image like the one shown here. It is easy to see that the Stx2 phage is present in the EHEC chromosomes (purple) and the outbreak genome (black), but not the EAEC or EPEC chromosomes.

*BRIG*, or the *BLAST Ring Image Generator*, is a Java-based tool for visualizing the comparison of a reference sequence to a set of query sequences [42,43]). Results are plotted as a series of rings, each representing a query sequence, which are coloured to indicate the presence of hits to the reference sequence (see Figure 4). *BRIG* is flexible and can be used to answer a broad range of comparative questions, depending on the selection of the reference and comparison sequences. However it is important to keep in mind that this particular approach is reference-based, meaning it can show you which regions of the reference sequence are present or absent in query sequences, but it cannot reveal regions of the query sequences that are missing from the reference sequence. Therefore the selection of the reference is critical to understanding the results. An example is given in Figure 4, in which an EHEC genome is used as the reference sequence and the *E. coli* O104:H4 outbreak genome assembly, along with other pathogenic *E. coli* genomes, are used as queries. This makes it easy to see that the outbreak strain differs significantly from enterohaemorrhagic *E. coli* (EHEC) in terms of gene content, but shares with it the Stx2 phage sequence which is missing from enteroaggregative *E. coli* (EAEC) and enteropathogenic *E. coli* (EPEC) (highlighted in Figure 4). The tutorial includes a second example, using the *E. coli* O104:H4 outbreak genome as the reference for comparison.

### Typing and public health applications: identifying resistance genes, sequence types, phage, plasmids and other specific sequences

Whole genome sequencing is increasingly being used in place of PCR-based sequencing or typing methods. Here we outline some specialist tools for these purposes. The tutorial contains instructions for using these tools to examine the *E. coli* O104:H4 outbreak genome.

The detection of antimicrobial resistance genes is a key question for many researchers, especially in public health and diagnostic labs. The *ResFinder* tool [44], freely available online [45], allows users to upload sequence data to search against its curated database of acquired antimicrobial resistance genes. Sequence search is performed by *BLAST*, and the output is displayed in a table format that indicates which resistance genes were found, where they were found (contig name and coordinates), and the expected effect on phenotype. The fastest way to use *ResFinder* is to upload a genome assembly, however it is also possible to upload raw sequence reads in fastq format, which will be assembled prior to searching for resistance genes.

Multi-locus sequence typing (MLST) is a widely used, sequence-based method for typing of bacterial species and plasmids [46]. In February 2013, public MLST schemes were available for over 100 bacterial species and five plasmid incompatibility types [47]. The Center for Genomic Epidemiology hosts a publicly available web-based tool [48] that allows users to upload sequence data and extract sequence types for most of the publicly available MLST schemes. Like *ResFinder*, the tool uses BLAST searches of assemblies to identify sequence types, and can accept either genome assemblies or read sets, which are assembled on the fly prior to searching. Sequence types can also be extracted directly from reads, which can be more sensitive than assembly; see e.g. *SRST*, a command-line tool based on read mapping [49,50].

For many bacteria, phage are the most dynamic part of the genome and are therefore of key interest to many researchers. Several free online tools exist for the identification of prophage sequences within bacterial genomes. A particularly feature-rich tool is *PHAST (PHAge Search Tool)*[51]. Genome assemblies can be uploaded in fasta or GenBank format; outputs include summary tables (indicating the location and identity of phage sequences within the assembly) and interactive tools for visualization of both the individual phage annotations and their locations on a circular map of the genome.

In most bacterial genome sequencing experiments, whole genomic DNA is extracted from the isolate and thus the sequence data includes both chromosomal and plasmid DNA. Many researchers are interested in exploring which plasmids are present in their bacterial genomes, particularly in the context of plasmid-borne resistance genes or virulence genes. One approach to rapidly detecting the presence and sequence type of a particular plasmid incompatibility group is to run a plasmid MLST analysis, *e.g.* using *SRST*[49]. However this will only work for the small number of plasmids with MLST schemes, and does not tell you which genes are encoded in the plasmid.

The ability to determine which sequences belong to plasmids and which belong to chromosomes varies with each sequencing experiment. This generally hinges on whether it is possible to assemble whole plasmids into a single sequence, which depends on many factors including read length, the availability of paired-end or mate-pair data, and the presence of repetitive DNA within the plasmid sequence. In most cases it is not possible to confidently assign every single contig to its correct replicon (*i.e.* chromosome or specific plasmid), without performing additional laboratory experiments. However, it is possible to get a very good idea of what plasmids are present in a genome assembly using comparative analyses. A good place to start is to identify all the contigs that are not definitely chromosomal (by comparing to other sequenced chromosomes using *ACT* or *Mauve*, see above) and *BLAST* these against GenBank or a plasmid-specific database. One such database is available on the PATRIC website [52]. On the PATRIC *BLAST* page, select ‘blastn’ from the Program dropdown list and select ‘Plasmid sequences’ from the Database dropdown list. At the bottom of the page you can choose to view your results graphically (great if you are just searching a few contigs) or as a table (better if you have lots of contigs to investigate). The most similar plasmid sequences should make good candidates for more detailed comparison and visualization using *Mauve*, *ACT* or *BRIG* as outlined above.

Another useful approach is to perform a *blastn* (nucleotide *BLAST*) search of the whole database at NCBI to see which known sequences your non-chromosomal contigs match (go to [53] and click ‘nucleotide blast’, then upload your contigs and make sure you are searching the ‘nr’ database). If you find you have a large contig with lots of matches to plasmid sequences, it’s likely your contig is also part of a plasmid. One advantage of using NCBI’s BLAST search page is that results can be viewed in the form of a phylogenetic tree (click ‘Distance tree of results’ at the top of the results page). This can help to quickly identify the plasmid sequences closest to yours, which can then be used for comparative analysis. If you find a contig that has close matches to part of a known plasmid, it may be of interest to know if the rest of the reference plasmid sequence is also present in the novel genome. You could get a quick idea of this using *BRIG* - use the known plasmid sequence as the reference and your set of assembled contigs as the query, then look to see how much of the known plasmid is covered by contigs. If more of the plasmid is covered, an ACT comparison could be performed using the reference plasmid and the annotated contig set, in order to identify which other contigs are likely to ‘belong’ to the same plasmid replicon and inspect what other genes are carried by the new plasmid.

### Other analyses

There are many other methods for performing comparative bacterial genomic analysis, which are not discussed here. In particular, we have not discussed phylogenetic analysis, or how to perform detailed gene content comparisons between sets of genomes.

Arguably, phylogenetic analysis of closely related genomes is best performed using single nucleotide polymorphisms (SNPs) identified by read mapping rather than assembly-based approaches [6,54,55]. Many software programs are available for this task; see [56,57] for a review and the updated software list hosted by the SeqAnswers web forum [58]. The process can be somewhat automated using command-driven pipelines such as *Nesoni*[59] or graphical-interfaces within the MiSeq or Ion Torrent analysis suites or the web-based *Galaxy*[60].

Detailed gene content comparisons are generally best-performed using databases tailored to the bacterial species of interest. An excellent place to start is to explore the web-based tools PATRIC [61] and PGAT [62], which are suitable for biologists with little or no programming skills.

### Delving deeper into bioinformatics

For biologists interested in learning more about bioinformatics analysis, we recommend two things. First, get comfortable with the Unix command-line [63,64], which opens up a huge array of software tools to do more sophisticated analyses (see [58] for a list of available next-generation sequence analysis tools). Second, learn to use the Python scripting language (tutorial at [65]) and associated BioPython functions [66], which will help you to write your own snippets of code to do exactly the analysis you want.

## Conclusions

The bench-top sequencing revolution has led to a ‘democratization’ of sequencing, meaning most research laboratories can afford to sequence whole bacterial genomes when their work demands it. However analysing the data is now a major bottleneck for most laboratories. We have provided a starting point for biologists to quickly begin working with their own bacterial genome data, without investing money in expensive software or training courses. The figures show examples of what can be achieved with the tools presented, and the accompanying tutorial gives step-by-step instructions for each kind of analysis.

## Competing interests

The authors declare that they have no competing interests.

## Authors’ contributions

DJE and KEH drafted, read and approved the manuscript.

## Supplementary Material

Additional file 1 **Tutorial. **Bacterial Comparative Genomics Tutorial. Detailed tutorial including worked examples, divided into three sections (**1**) Genome assembly and annotation, (**2**) Comparative genome analysis, and (**3**) Typing and specialist tools. Click here for file
