# Supplementary material for: Beginner’s guide to comparative bacterial genome analysis using next-generation sequence data
Source: Microb Inform Exp. 2013 Apr 10;3:2. doi: 10.1186/2042-5783-3-2 (PMC3630013; doi:10.1186/2042-5783-3-2)
Supplement: Additional file 1 — Tutorial. Bacterial Comparative Genomics Tutorial. Detailed tutorial including worked examples, divided into three sections (1) Genome assembly and annotation, (2) Comparative genome analysis, and (3) Typing and specialist tools. [file 2042-5783-3-2-S1.pdf]

Bacterial Comparative Genomics Tutorial

Accompanying the paper:

Beginner's guide to comparative bacterial genome analysis using next-generation sequence data
David J. Edwards, Kathryn E. Holt

BMC Microbial Informatics, 2013

Last updated March 2013

1. Genome assembly and annotation 2
1.1 Downloading *E. coli* sequences for assembly 2
1.2 Examining quality of reads (FastQC) 2
1.3 Velvet – assembling reads into contigs 4
1.3.1 Using VelvetOptimiser to optimise *de novo* assembly with Velvet 6
1.4 Ordering contigs against a reference using Mauve 7
1.4.1 Viewing the ordered contigs (Mauve) 10
1.4.2 Viewing the ordered contigs (ACT) 13
1.5 Mauve Assembly Metrics – Statistical View of the Contigs 15
1.6 Annotation with RAST 15
1.6.1 Alternatives to RAST 19
2. Comparative genome analysis 20
2.1 Downloading *E. coli* genome sequences for comparative analysis 20
2.2 Mauve – for multiple genome alignment 21
2.3 ACT – for detailed pairwise genome comparisons 24
2.3.1 Generating comparison files for ACT 24
2.3.2 Viewing genome comparisons in ACT 27
2.4 BRIG – Visualizing reference-based comparisons of multiple sequences 29
3. Typing and specialist tools 34
3.1 PHAST – for identification of phage sequences 34
3.2 ResFinder – for identification of resistance gene sequences 34
3.3 Multilocus sequence typing 34
3.4 PATRIC – online genome comparison tool 34

1. Genome assembly and annotation

1.1 Downloading *E. coli* sequences for assembly

In this part of the tutorial, we will create a draft quality *E. coli* O14:H4 genome assembly to use in the comparative genome analysis. To start with we need sequences to assemble. For the worked example we are using Illumina HiSeq paired-end reads from *E. coli* O104:H4 strain TY-2482 (ENA accession SRR292770) – available here http://www.ebi.ac.uk/ena/data/view/SRR292770&display=html. Locate the 'Fastq files (ftp)' column and right-click on each of the two file links, choosing 'Save Link as…' to save them to your computer. These are in fastq format (see http://en.wikipedia.org/wiki/FASTQ_format) and compressed using gzip (you do not need to uncompress them).

Remember to download both forward and reverse reads (named 'SRR292770_1.fastq.gz' and 'SRR292770_2.fastq.gz'). Save these to a new folder (directory) with a suitable name, e.g. 'comparison_tut'. This will be our working directory for the tutorial.

1.2 Examining quality of reads (FastQC)

Prior to attempting to assemble a set of reads, it is good practice to examine the reads to see if they are of good quality. A simple package to install and run to examine reads is FastQC.

Website: Download and install FastQC from http://www.bioinformatics.babraham.ac.uk/projects/fastqc/.
The website also features examples of good and poor quality read sets for a number of sequencing platforms.

Compatibility: Java based, available for Windows, Linux and Mac OS X.
This tutorial was created using FastQC 0.10.1 on Mac OS X. Some versions of Java have been disabled on the Mac OS X, and if you do not have a version of Java higher than version 7u11 installed, you may need to follow the suggestions in the FAQ for Java (http://www.java.com/en/download/faq/java_mac.xml).

Inputs: forward and reverse read sequence files (fastq format)

Instructions

Once FastQC has been installed, open the program to begin. Then:

1. To select the file sequence to check, use 'File > Open' in the FastQC menu. Navigate to the folder that you put the TY-2482 reads and select the 'SRR292770_1.fastq.gz' file. Make sure 'File Format' is set to

'Sequence Files', then hit the 'Open' button. FastQC will commence the analysis.

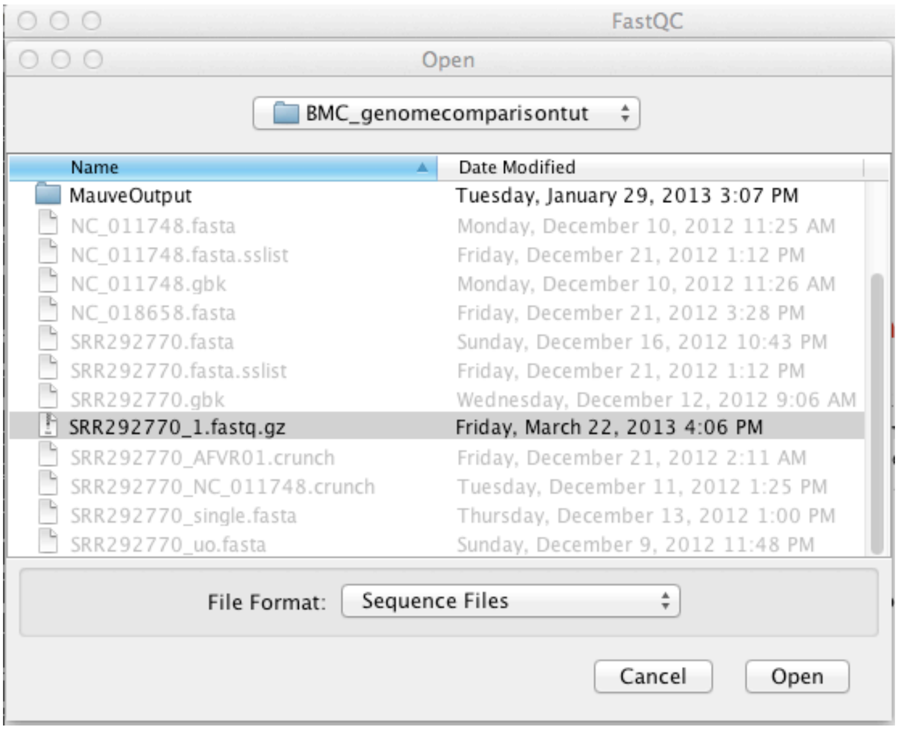

2. When the analysis has finished, you will be presented with a series of reports on the sequences. Select 'Per base sequence quality' to see a graph of the same. It should look like this:

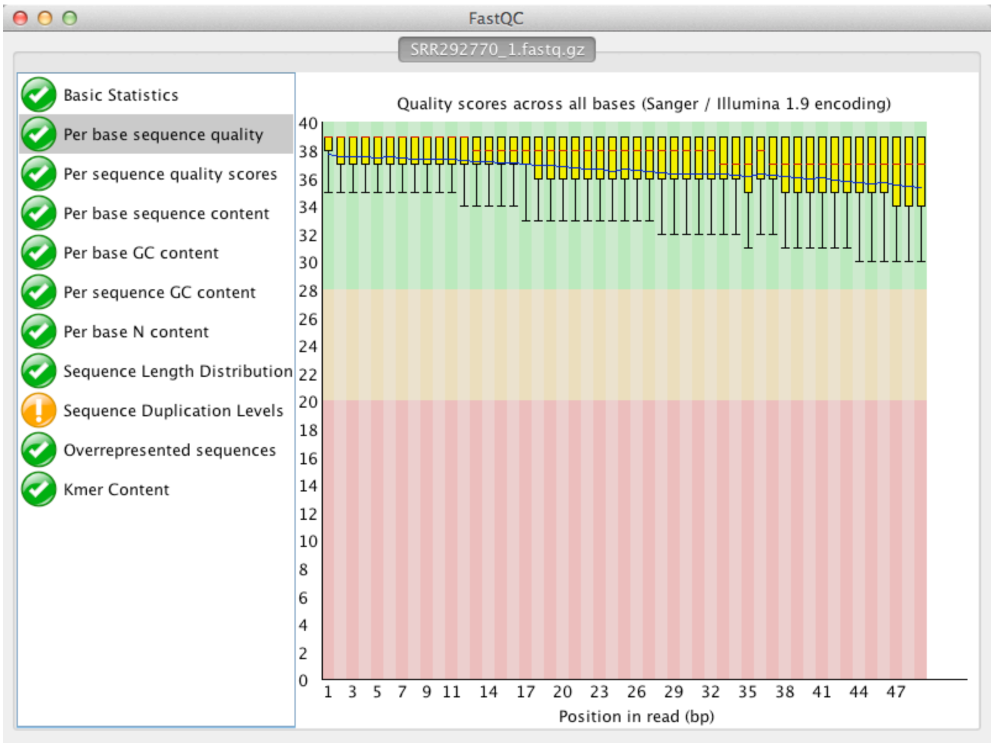

You can also examine the other reports.

Note that this sequence set passes most of the tests, though the sequence duplication level is a little high (around 26%). The assembly could be improved by first removing duplicates by making use of a fastq quality control package such as the command line tools FASTX-Toolkit (http://hannonlab.cshl.edu/fastx_toolkit/) or Trimmomatic (http://www.usadellab.org/cms/index.php?page=trimmomatic). However, as the reads for the tutorial are of otherwise good quality, we shall leave the important topic of quality control, and its pit-falls, for others to describe. The websites for the two packages are a good place to start, along with the supporting information for FastQC.

You can now close FastQC and continue with the rest of the tutorial. If you wish to save the report beforehand, use 'File > Save report...' before closing.

1.3 Velvet – assembling reads into contigs

Website: Download and install Velvet and its manual (~25 MB) from http://www.ebi.ac.uk/~zerbino/velvet/

Compatibility: Can be compiled for Windows, Mac OS X and Linux, though a 64-bit environment and a minimum of 4GB of RAM are recommended.
This tutorial was created using Velvet 1.2.08 on Mac OS X.

Reference: Zerbino, D. R. and Birney, E., Velvet: algorithms for de novo short read assembly using de Bruijn graphs. *Genome Res*, 2008. gr.074492.107 [pii] 10.1101/gr.074492.107.

Instructional Reference: Zerbino, D. R., Using the Velvet de novo assembler for short-read sequencing technologies. *Current protocols in bioinformatics / editoral board, Andreas D. Baxevanis ... [et al.]*, 2010. 10.1002/0471250953.bi1105s31.

Inputs: forward and reverse read sequence files (fastq format)

Instructions

The *de novo* assembly program Velvet we used was installed with the 'MAXKMERLENGTH' set at 101 bp (`make 'MAXKMERLENGTH=101'`) – see the Velvet manual for more details. Note that a maximum *k*-mer of 41 will be sufficient for this exercise, but longer *k*-mers are required when working with longer HiSeq and MiSeq generated reads (which are now typically >100 bp). Note you will also need to add the Velvet directory to your path, or use the full path to the 'velvetg' and 'velveth' executables in the commands below.

1. Open a terminal session and change the directory to that containing the SRR292770 reads files:

```
cd comparison_tut
```

2. First we need to run velveth, enter:

```
velveth out_data_35 35 -fastq.gz -shortPaired -separate
SRR292770_1.fastq.gz SRR292770_2.fastq.gz
```

This will take ~1-2 minutes and will produce a hash table of the reads using the specified *k*-mer length (k=35), saving them to the folder 'out_data_35'. The –shortPaired and –separate tag tells Velvet we are supplying short, paired end reads with separate files for forward and reverse reads. See manual for other input options.

3. The next Velvet step to run is velvetg to build the graph. Enter:

   ```
   velvetg out_data_35 -clean yes -exp_cov 21 -cov_cutoff
   2.81 -min_contig_lgth 200
   ```

   This will take ~5 minutes. Running this command will output a number of files to the same folder as velveth, including the file containing our newly assembled contigs – this will be labelled 'contigs.fa'. Minimum contig length is set to 200bp as this is the shortest length allowed for GenBank submission of draft genomes. The coverage cut-offs specified here are ones we have pre-determined to be optimal for assembly of this read set. See below for info on using VelvetOptimiser to set cut-offs for different read sets.

4. Copy the contigs file from the Velvet output folder and rename it:

   ```
   cp out_data_35/contigs.fa SRR292770_unordered.fasta
   ```

   You can then delete the output folder 'out-data-35', though you may want to either save or look at the statistic file, 'stats.txt', before doing so.

Whilst we provide 'optimal' values for the three options of Velvet (*k*-mer = 35, expected coverage = 20, coverage cutoff of 2.81), these can be changed to examine how each affects the contigs produced. Note: you can rerun just the velvetg command with new values if you are varying only the latter two and keeping the *k*-mer constant by <u>keeping</u> the Velvet output folder between runs of velvetg.

1.3.1 Using VelvetOptimiser to optimise *de novo* assembly with Velvet

To get the 'optimal' values used here, we made use of the Perl script VelvetOptimiser (we used version 2.2.5) available for download at http://bioinformatics.net.au/software.velvetoptimiser.shtml. Here, we provide instructions for running VelvetOptimiser to demonstrate how these values were obtained, and for those interested in doing the same – we include it as a further exercise in making use of Velvet. Those interested in exploring both even further should begin with the instructional paper by Zerbino (2010). (Those not yet comfortable with Unix, Perl and the command line may wish to skip the following.)

In order to run VelvetOptimiser, you will also need to download and install both Perl (version 5.8 or later, http://www.perl.org/) and BioPerl (version 1.4 or later, http://www.bioperl.org/wiki/Main_Page). Obviously, you also need Velvet as above.

1. Open a terminal session and change to the directory containing the reads files.

2. To run VelvetOptimiser, enter:

```
VelvetOptimiser.pl -s 33 -e 41 -f '-fastq.gz -shortPaired
-separate SRR292770_1.fastq.gz SRR292770_2.fastq.gz' -o
'-min_contig_lgth 200' -p SRR292770
```

With these settings, VelvetOptimiser will set up a series of velveth runs using odd-number kmers between 33 and 41. It then runs velvetg for each, taking the one with the best N50 as the seed for the final optimisation of the coverage cutoff, where the number of bases in contigs longer than 100bp is used as the optimising statistic. The output is the same as for a regular Velvet run, though the output folder will have the prefix 'SRR292770' to keep it separate from the earlier Velvet run described above. The logfile for the run (SRR292770_logfile.txt) contains details of the run, including the commands used to run velveth and velvetg.

For those interested in assembling Ion Torrent sequence reads, we recommend you try MIRA (version 3, http://www.chevreux.org/projects_mira.html). This assembler is also useful for those interested in assembling reads from different sequencing technologies into the one assembly – MIRA is probably the best for this kind of assembly project. Once you have assembled the reads into contigs using MIRA, the rest of the analysis can make use of the tools and methods described here for Illumina-based reads.

1.4 Ordering contigs against a reference using Mauve

Once the sequence reads have been assembled into contigs, it is useful to order them against a suitable reference genome. One simple way to accomplish this is to use the 'Move Contigs' option available in *Mauve* (which is also used below for genome comparisons).

Website: http://asap.ahabs.wisc.edu/mauve/ (Includes download links, installation instructions and user guide)

Compatibility: Java based, available for Windows, Mac OS X, and Linux
This tutorial was created using Mauve 2.3.1 on Mac OS X.

Reference: Darling, A. E., Mau, B. and Perna, N. T., "progressiveMauve: multiple genome alignment with gene gain, loss and rearrangement". *PLoS One*, 2010 5(6): e11147.

Inputs: These will be your newly assembled contigs and a reference genome – here we have chosen to use Ec55989 (NCBI accession NC_011748), a closely-related strain with a complete genome, available for download from NCBI. Go to this link:
ftp://ftp.ncbi.nih.gov/genomes/Bacteria/Escherichia_coli_55989_uid59383/
And download the sequence in fasta format, NC_011748.fna (right-click to save to your computer).

If you don't want to run your own Velvet assembly, you can do the rest of the exercises using pre-assembled *E. coli* O104:H4 contigs. Go to http://www.ncbi.nlm.nih.gov/Traces/wgs/?val=AFVS01 and click the 'Download' tab, then right-click the fasta file to your computer and unzip it.

Instructions

Once you have installed Mauve and located your reference genome and contigs, we can order the contigs.

1. Launch the Mauve application.

2. From the Tools menu, select 'Move Contigs'.

3. A dialogue box should appear, with a box labelled 'Choose location to keep output files and folders'. Navigate to the folder with the sequences and the copied contigs, then click the 'Create New Folder' radio button. Give this folder a suitable name, *e.g.* 'MauveOutput' and then hit 'OK'.

4. A message should appear telling you about the iterative process involved in reordering the contigs. Take note of it, then hit 'OK' to dismiss it.

5. A dialogue box should appear, with a box labelled 'Align and Reorder Contigs'. Click the button below the box 'Add Sequence…' and navigate to the reference genome to align against, in this case 'NC_011748.fna'.

6. Click the 'Add Sequence…' button again and navigate to the fasta file of the contigs you wish to align, 'SRR292770_unordered.fasta' from the assembly exercise above. Check that you have put the reference genome first, and the draft second, as expected by Mauve.

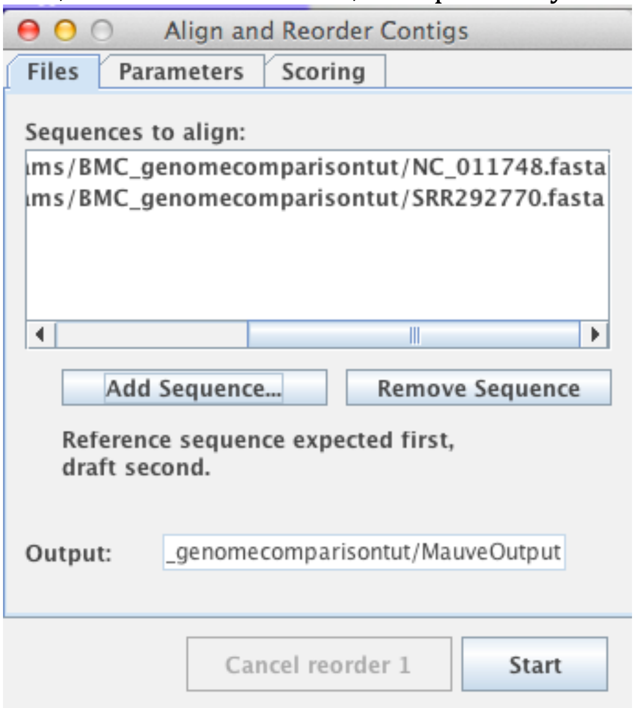

7. Click 'Start' to run the reordering. This might take half an hour or so total. A new window should appear marked 'Mauve Console' where the progress of the run will be displayed, including any error messages (see below for an example). The reordering will take from four to seven iterations (for Mac OS X; even up to 16 iterations and a bit more time on a 32-bit Windows OS). A new window of the visualization tool should launch for each completed iteration, marked 'Mauve unknown – alignmentX', where X is the iteration number.

   If you encounter errors, check that you have specified the right files for input – they should be fasta or multi-fasta sequence files.

8. Finally, a message telling you the reorder is completed should appear. Hit 'OK' and quit Mauve – though you can inspect the final alignment (and the others) beforehand.

9. The final set of ordered and oriented contigs are in the fasta file located in the last of the iterated alignments. To find it, look in the 'MauveOutput' folder created above. For each iteration of the reordering there will be an output folder, so the final output is the contig file located in the subdirectory 'alignmentX' with the highest X, where X is the iteration number. Rename 'SRR292770_unordered.fasta' in this subdirectory, to 'SRR292770.fasta' and copy it to your main working directory (*i.e.* the one with the original sequence files, make sure you have changed the name of

the ordered contigs file first as we will use the unordered contigs in a later exercise, *e.g.* 'SRR292770_unordered.fasta'. You can then delete the 'alignmentX' folders.

Those who are used to Unix and sequence analysis may prefer to use a command-line based solution for ordering contigs. We recommend Abacas (http://abacas.sourceforge.net/index.html), which requires installation of MUMmer (http://mummer.sourceforge.net/), Perl and BioPerl.

The command for ordering against a reference genome is (assuming you have attached Abacas [version 1.3.1] to the $path environment and renamed the contigs file from the first exercise to 'SRR292770_unordered.fasta' first):

```
abacas.1.3.1.pl –r NC_011748.fasta -q
SRR292770_unordered.fasta –p 'nucmer' –c –m –b –o
SRR292770.fasta
```

Using either method, you should end up with a set of contigs ordered against the reference strain in multi-fasta format in a file called 'SRR292770.fasta'. This is the file to use for the following steps.

1.4.1 Viewing the ordered contigs (Mauve)

To examine the newly ordered contigs, we provide two GUI-based approaches. For the first, both the program Mauve and instructions for the comparison method are as detailed below, albeit with a few minor (but important) changes.

In this example, we will generate a multiple alignment of the ordered contigs from the O104:H4 outbreak genome, the Ec55989 genome used as the reference for ordering, and another assembly created using more read sets than our draft genome, and a different assembler. This alternative assembly of strain TY-2482 (NCBI accession AFVR01) is available for download here http://www.ncbi.nlm.nih.gov/Traces/wgs/?val=AFVR01 in fastq gzip format *via* the download tab. After downloading, unzip the file before continuing. **Order this alternative assembly to the Ec55989 reference genome first – use the instructions provided above.**

Instructions:

1. Launch the Mauve application

2. From the File menu, select 'Align with progressiveMauve…'

3. A dialogue box should appear, with a box labelled 'Sequences to align:'. Click the button below the box 'Add Sequence…' and navigate to your ordered contigs file, 'SRR292770.fasta'.

4. Click the 'Add Sequence…' button again and navigate to the fasta file of a genome you wish to align. In this case, we will start with the alternative assembly, 'AFVR01.fasta'. If you provide a multi-fasta file containing contigs, Mauve will concatenate these together before running the alignment.

5. Repeat step 4 to add any other sequences of interest. In our example we will just add the EAEC genome Ec55989.

6. Now we need to specify the output file. Click the button marked '…' to select an output file. Navigate to the directory in which you want the output to appear. Now specify a name for the output file (e.g. 'mauve_output'), and click 'Save'.

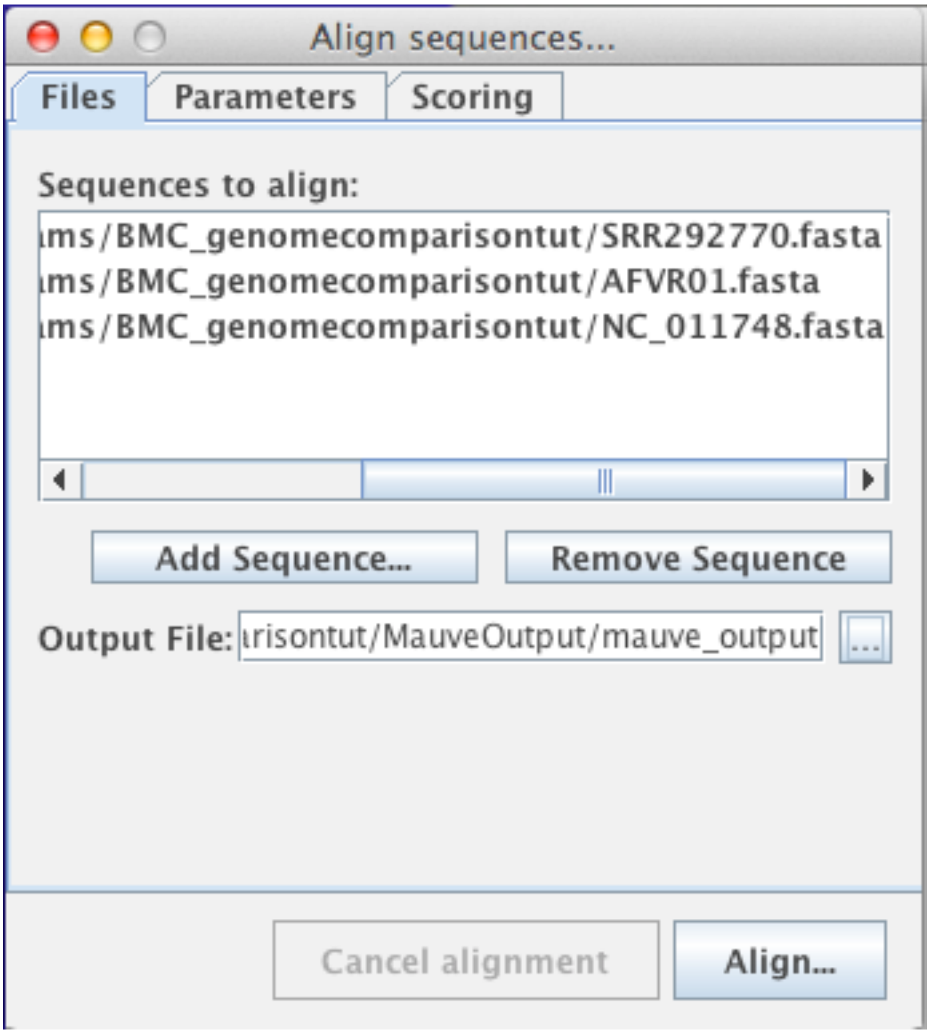

7. Click 'Align...' to run the alignment. This might take half an hour or so. A new window should appear marked 'Mauve Console' where the progress of the run will be displayed, including any error messages (example below).

   If you encounter errors, check that you have specified the right files for input – they should all be fasta or multi-fasta sequence files, and can include up to one genome in Genbank format (to provide an annotation).

8. When the alignment is finished, the visualization tool will appear. To simplify the image a little, select View -> Style -> uncheck 'LCB connecting lines'. It should look like this:

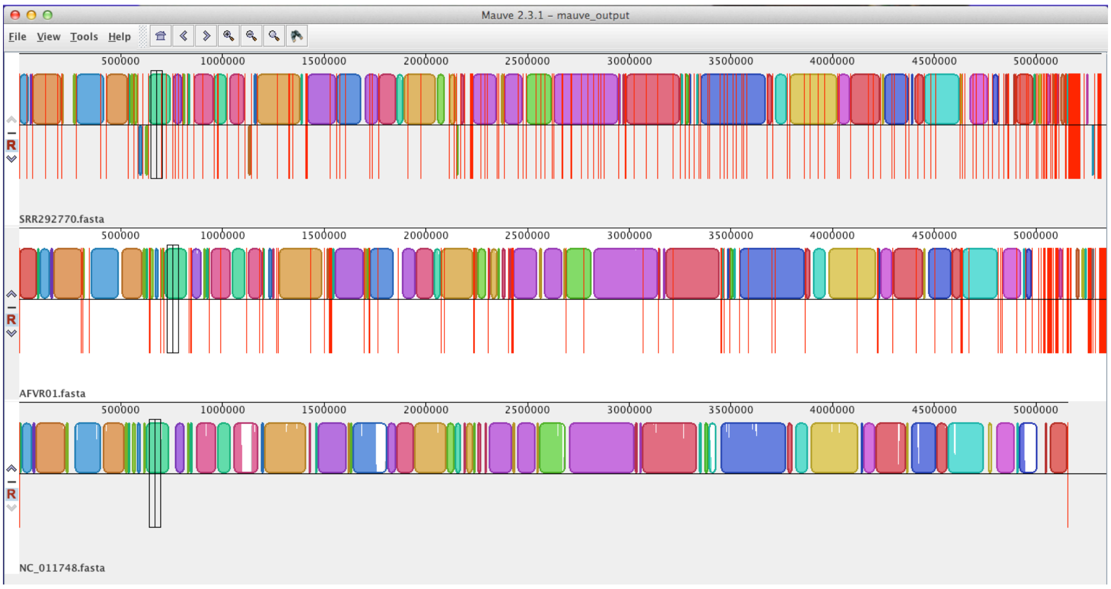

Row 1 = O104 ordered contigs.
Row 2 = alternative assembly
Row 3 = Ec55989 (EAEC) genome
Coloured blocks indicate regions of sequence with homology in the other genomes.
Red lines indicate contig boundaries.

Notice the similarity in the orders of our Velvet assembly and the alternative assembly. Both assemblies contain contigs that don't map to the reference genome.

You can save a static image of what you are viewing by selecting Tools -> Export -> Export image…

1.4.2 Viewing the ordered contigs (ACT)

We will now use ACT to compare the same three genomes, our O104:H4 assembly, the alternative assembly and the reference genome Ec55989. Note both assemblies should have been ordered against Ec55989 as outlined above.

Details of downloading and using ACT are given below (2.3.1).

Inputs: ACT can display pairwise comparisons between genomes. To do this it needs the genome sequences themselves (in fasta format or annotated sequence format such as Genbank or EMBL files) and a comparison file. Comparison files can be created on your computer if you have BLAST installed, or using an online tool like WebACT (http://www.webact.org/) or DoubleACT (http://www.hpa-bioinfotools.org.uk/pise/double_act.html), see steps 1-2 below.

Instructions

Use the instructions for using ACT below to:

1. Generate first a single fasta file for the two assemblies (step 1, 2.3.1).
2. Generate a comparison file for our O104:H4 assembly against both Ec55989 and the alternative assembly separately. (step 2, 2.3.1)
3. View the comparison(s) in ACT.
   a. Launch the ACT application
   b. Select File -> Open
   c. Initially, boxes for 2 sequence files and 1 comparison file will be displayed. Click 'more files…' to create boxes for a second comparison file and a third sequence file.
   d. Click the 'Choose…' buttons to select each of your two sequence files and your comparison file. Note that you can load in your multi-fasta contigs files at this point for the *E. coli* O104:H4 and alternative assembly. We want the *E. coli* O104:H4 assembly in the middle, with the comparisons to Ec55989 and the alternative assembly above and below it, like this:

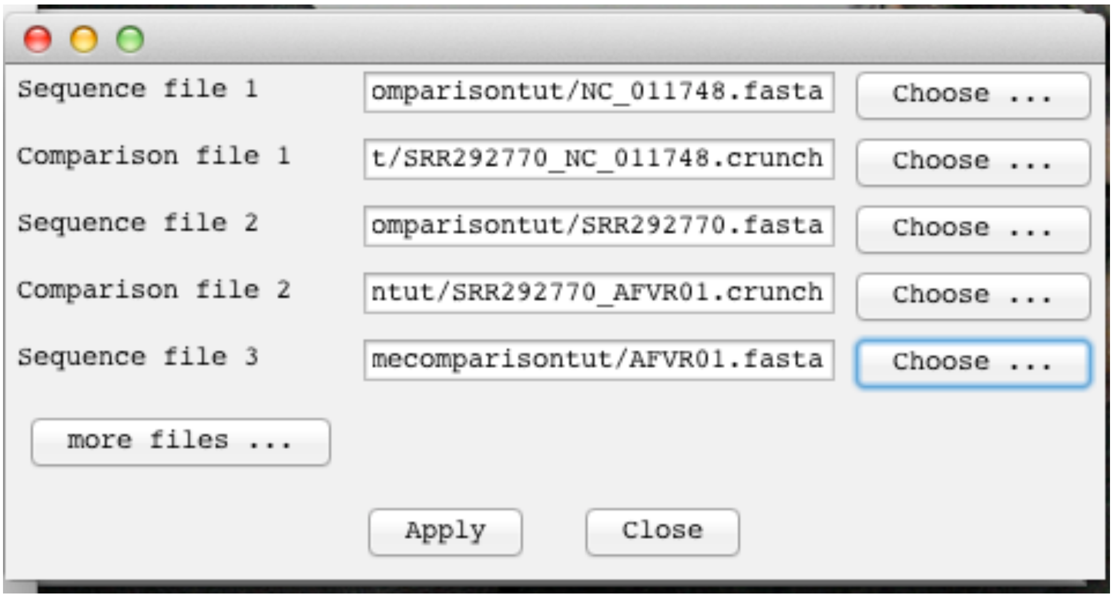

e. The comparison between the three genomes will be displayed. See the ACT manual for details of how to navigate around the viewer. Here, we are comparing the new *E. coli* O104:H4 genome assembly (middle) with Ec55989 (top) and the alternative assembly (bottom). We have zoomed out by clicking the down arrow at the bottom right of the window. Since our contigs were ordered against the Ec55989 genome, all the *E. coli* O104:H4 contigs with no homology to Ec55989 (*i.e.* no coloured bars linking them to Ec55989) appear at the end of the sequence. Some of these contigs do map to the genome of the alternative assembly. Also note that there is much higher homology between our O104 assembly and the alternative one.

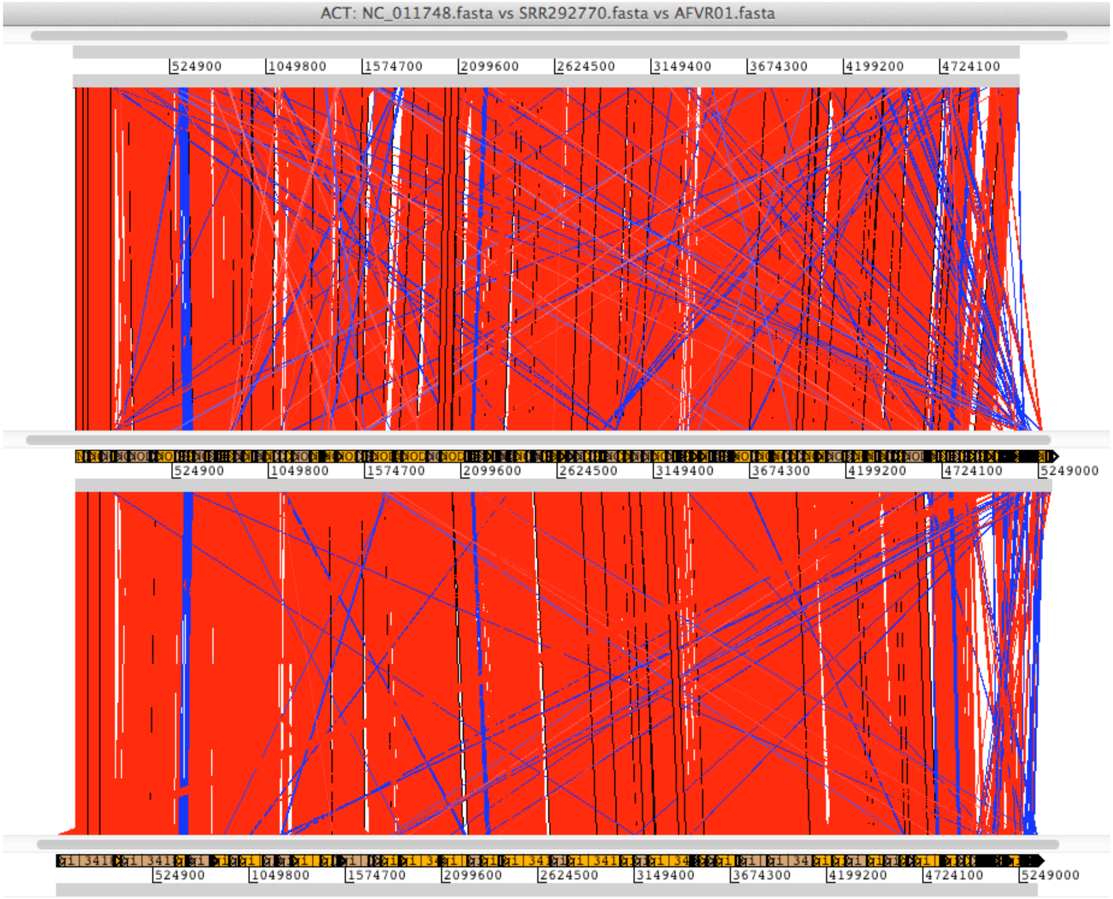

1.5 Mauve Assembly Metrics – Statistical View of the Contigs

Website:
http://code.google.com/p/ngopt/wiki/How_To_Score_Genome_Assemblies_with_Mauve

Compatibility: Available for Windows, Mac OS X, and Linux versions of Mauve, but see the text for more details. It also requires the R statistical program to be installed. See above link for more details.

Reference: 1. Darling, A. E., *et al.*, "Mauve assembly metrics." *Bioinformatics*, 2011. 10.1093/bioinformatics/btr451.

Inputs: Ordered contigs file (multi-fasta format), reference genome (more closely related the better)

Installation note: The authors indicate that it is possible to use Mauve Assembly Metrics via the Mauve GUI tool when only a single pairwise comparison is run, but as they do not provide specific instructions, we can only describe how to do so for the Mac OS X. The following may also work for the Linux version, but has not been tested by us. Unfortunately, we do not yet have a solution for installing Mauve Assembly Metrics in the Windows-based version of the Mauve GUI.

The simplest way of installing Mauve Assembly Metrics into the GUI tool of Mauve for the Mac OS X is to use the instructions for installing Mauve Assembly Metrics by script (see the above website for details) with one important change – edit the target '.dmg' file to the most current update from the Mauve download website. You may still have to install Mauve as an application by 'drag-and-drop'.

Mauve Assembly Metrics are only available as a GUI tool for single pairwise comparisons, *e.g.* between the reference genome and the assembly as ordered contigs. You will know if the tool is installed successfully if a new button appears after running such a comparison, as highlighted here with the red circle:

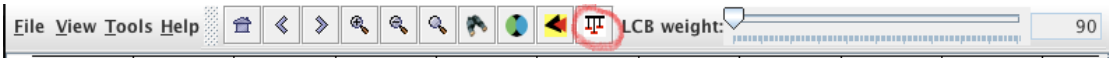

Instructions
In this example, we will generate Mauve Assembly Metrics for the assembly we created using a complete genome from the outbreak, *E. coli* O104:H4 strain 2011C-3493 (NCBI accession NC_018658.1; download NC_018658.fna from ftp://ftp.ncbi.nih.gov/genomes/Bacteria/Escherichia_coli_O104_H4_2011C_3493_uid176127/)

1. Using the instructions above, reorder the SRR292770 unordered contigs to the new reference, 2011C-3493.

2. When the alignment has finished, don't close Mauve but instead, on the final alignment window hit the Mauve Assembly Metrics button. This will launch the report window and should look like this:

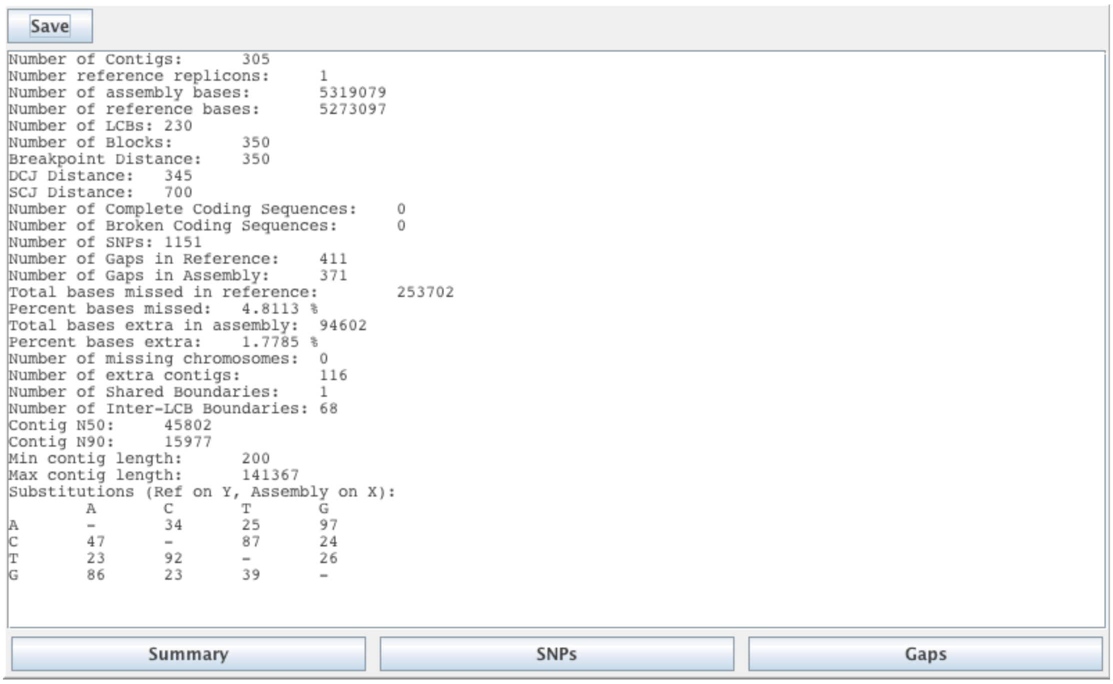

Notice that along with the summary shown here, you can also generate a report of SNPs and gaps in alignment, and save these reports. To understand the report fully, read the reference for Mauve Assembly Metrics given above.

Some highlights from our assembly include an N50 of 45,802 bp, with a largest contig of 141,367 bp and the smallest of 200 bp (as expected, as we set that in Velvet). Notably almost 5% of bases of the reference genome have been missed, though our assembly has an extra 2% of bases, making up an extra 116 (small) contigs that don't align. There are also some 1,150 SNPs between the two sequences, and interestingly, our assembly seemingly has fewer gaps (371) than the reference 2011C-3493 (411).

Mauve Assembly Metrics can also be run as a command-line tool, with the instructions for installing and running the metrics tool provided at the same link as above. The advantage of the command-line version is that more than one assembly can be tested for inclusion in the same report output. As we are dealing with a single assembly in this tutorial, we leave this as an exercise for those so interested.

1.6 Annotation using RAST

Website: http://rast.nmpdr.org/ (need to register to use the service)

Compatibility: Most web browsers.
This tutorial was created using RAST version 4 on the Firefox web browser (version 17.0).

Reference: Aziz, R. K., *et al.*, "The RAST Server: rapid annotations using subsystems technology*." BMC Genomics*, 2008. 10.1186/1471-2164-9-75.

Inputs: Ordered contigs file (multi-fasta format)

Instructions
In this example, we will generate a GenBank annotation for the newly assembled and ordered contigs of the *E. coli* O104:H4 strain in multifasta format (use the contigs ordered against Ec55989, as we have done). **You must first register a for a RAST user account.**

1. Go to http://rast.nmpdr.org/ in a web browser and log into your account.

2. Under the 'Your Jobs' tab (top left corner) select 'Upload New Job'.

3. You should be taken to a page titled 'Upload your genome'. At the bottom of the page there is a box labelled 'File Upload:' click the button and navigate to your ordered contigs file ('SRR292770.fasta'). Then hit the 'Use this data and go to step 2' button. This may take a little while as it is uploading your sequence file over your internet connection.

4. Eventually the next page will open with the same heading as the last, with the sub-heading 'Review genome data', and some contig statistics. You will be asked to enter further details about the organism. In the first field, labelled 'Taxonomy ID', enter the code for *E. coli* (562), and hit the 'Look up taxonomy ID at NCBI' button. This will populate the rest of the fields for you, except the last, the stain. Enter 'TY-2482' into the space for the strain, and then hit the 'Use this data and go to step 3' button.

   (Note if you were doing this with something other than *E. coli*, you can find the right taxonomy ID at http://www.ncbi.nlm.nih.gov/taxonomy).

5. The next page should have sub-heading 'Complete Upload'. You can enter optional information (Sequencing Method = 'other', Coverage = '>8x', Number of contigs = "101-500"), but this is not necessary to use RAST. The other options for the RAST annotation pipeline should at least be considered, though we will use the default options as shown when the page first loads. Your final page should look like this:

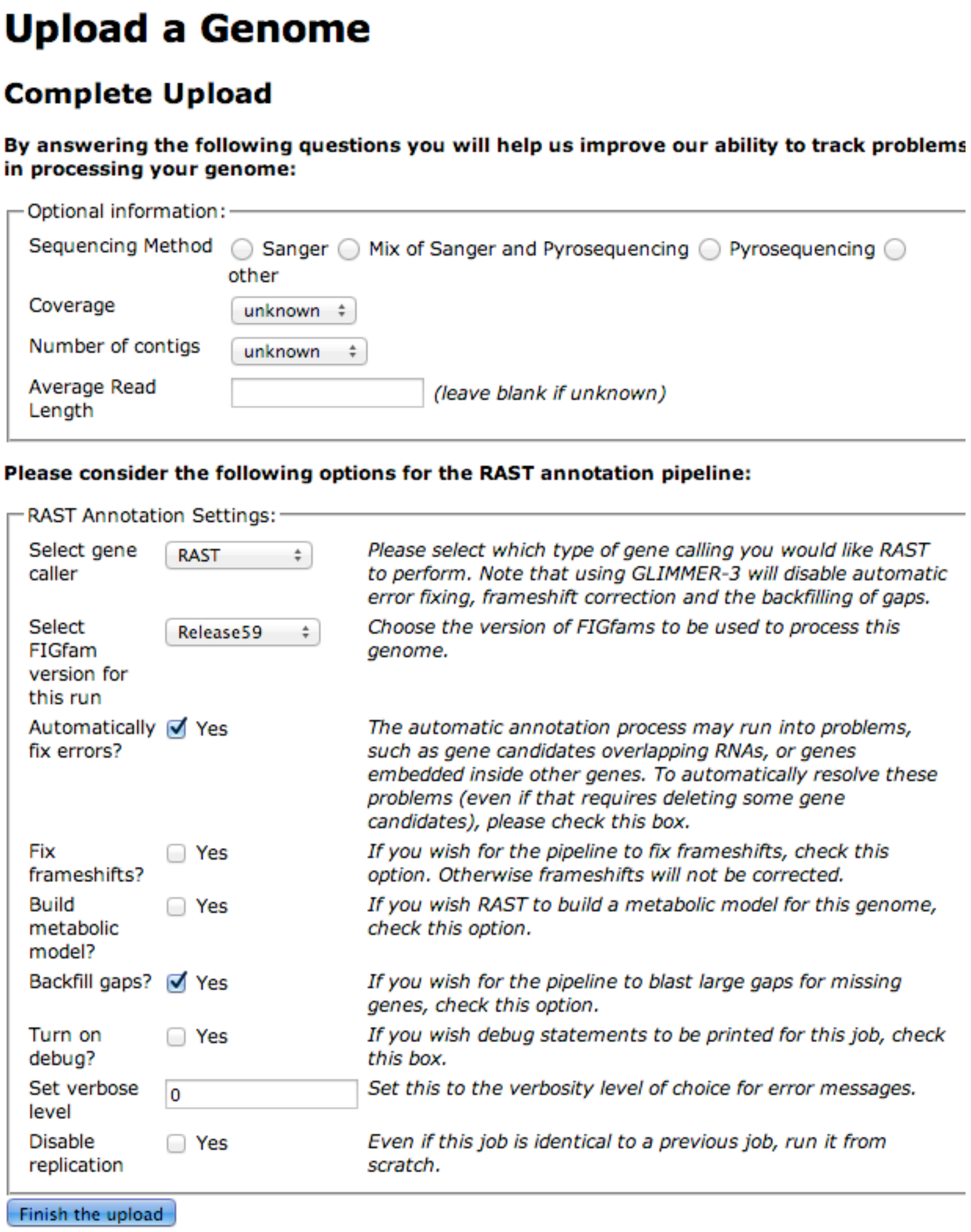

If it does, hit the 'Finish the upload' to start the job. Your job will join the submission queue, and you will be sent an email (to the address you used to register) when the job is completed. This could take a half a day or even much longer, depending on the number of jobs in the queue before you.

6. Once you receive the completion email from the Annotation Server, click on the link in the email to return to the RAST server (if you have logged out, you will have to log back in to continue). This time select 'Jobs Overview' under the 'Your Jobs' tab.

7. This will open the Jobs Overview page, where you will see a list of your jobs with a number of details and the status of the job. Click on the '[view details]' link for the job (in the 'Annotation Progress' column, under the green progress bars).

8. This opens the "Job Details' page and will include the available downloads if the job has completed. Select 'Genbank (EC numbers stripped)' and then hit 'Download'. The file will be call '562.<job_no.>.ec-stripped.gbk' – change this to 'SRR292770.gbk' and move the file to your work folder (where 'SRR292770.fasta' is located).

1.6.1 Alternatives to RAST

A number of command-line tools are available for annotation on a local machine. For fast *de novo* annotation we recommend trying Prokka (http://www.vicbioinformatics.com/software.prokka.shtml), though Prokka in turn relies on the installation of a long list of other programs (see the link for details). For those interested in comparative annotation, you could try BG7 (http://bg7.ohnosequences.com/). Otherwise, you now have an annotated draft genome for *E. coli* O104:H4 strain TY-2482, and can move on to the comparative genome analysis that follows.

2. Comparative genome analysis

2.1 Downloading *E. coli* genome sequences for comparative analysis

In this part of the tutorial, we will compare our *E. coli* O14:H4 genome assembly to other *E. coli* using various software packages on our computer. You will need to download the programs from the web using the links given in each section. In addition you will need to download some *E. coli* data for comparison.

For the Mauve and ACT comparisons, we will use these:

(This one we have already used above)
EAEC str. Ec55989 (NC_011748) – download NC_011748.fna from
ftp://ftp.ncbi.nih.gov/genomes/Bacteria/Escherichia_coli_55989_uid59383/

EHEC O157:H7 str. EDL933 (NC_002655) – download NC_002655.fna from
ftp://ftp.ncbi.nih.gov/genomes/Bacteria/Escherichia_coli_O157_H7_EDL933_uid57831/

To compare the O104:H4 genome to enterohaemorrhagic *E. coli* (EHEC) and enteropathogenic *E. coli* (EPEC), we will also use these genomes:

- EPEC O26:H11 str. 11368 - NC_013361.gbk from
ftp://ftp.ncbi.nih.gov/genomes/Bacteria/Escherichia_coli_O26_H11_11368_uid41021/
- EPEC O127:H6 str. E2348/69 - NC_011601.gbk from
ftp://ftp.ncbi.nih.gov/genomes/Bacteria/Escherichia_coli_O127_H6_E2348_69_uid59343/
- aEPEC O111:H9 str. E110019 - AAJW02.gbk from
http://www.ncbi.nlm.nih.gov/Traces/wgs/?val=AAJW02
- EHEC O157:H7 str. TW14359 - NC_013008.gbk from
ftp://ftp.ncbi.nih.gov/genomes/Bacteria/Escherichia_coli_O157_H7_TW14359_uid59235/
- EHEC O157:H7 str. EC4115 - NC_011353.gbk from
ftp://ftp.ncbi.nih.gov/genomes/Bacteria/Escherichia_coli_O157_H7_EC4115_uid59091/
- EHEC O157:H7 str. Sakai - NC_002695.gbk from
ftp://ftp.ncbi.nih.gov/genomes/Bacteria/Escherichia_coli_O157_H7_Sakai_uid57781/
- Stx (shiga-toxin, or verotoxin) prophage VT2
http://www.ncbi.nlm.nih.gov/nuccore/5881592
- LEE pathogenicity island
http://www.ncbi.nlm.nih.gov/nuccore/2897961

2.2 Mauve – for multiple genome alignment

We have already introduced Mauve above, for ordering contigs and inspecting assembly statistics.

In this example, we will generate a multiple alignment of the newly assembled and annotated O104:H4 outbreak genome (GenBank format) with an EHEC chromosome and the chromosome of EAEC strain Ec55989 (fasta). We will then view the alignment and use it to inspect genes that are annotated in the outbreak genome but missing from the other pathogen chromosomes.

Website: http://asap.ahabs.wisc.edu/mauve/ (Includes download links, installation instructions and user guide)

Compatibility: Java based, available for Windows, Mac OS X, and Linux
This tutorial was created using Mauve 2.3.1 on Mac OS X.

Reference: Darling, A. E., Mau, B. and Perna, N. T., "progressiveMauve: multiple genome alignment with gene gain, loss and rearrangement". *PLoS One*, 2010. 5(6): e11147

Inputs: Genome sequence files (fasta format) and up to one annotated genome sequence (Genbank format).

Instructions

1. Launch the Mauve application

2. From the File menu, select 'Align with progressiveMauve…'

3. A dialogue box should appear, with a box labelled 'Sequences to align:'. Click the button below the box 'Add Sequence…' and navigate to your annotated genome (GenBank file generated by RAST).

4. Click the 'Add Sequence…' button again and navigate to the fasta file of a genome you wish to align. In this case, we will start with the genome of the EHEC O157:H7 strain EDL933 (NC_002655.fna). If you provide a multi-fasta file containing contigs, Mauve will concatenate these together before running the alignment.

5. Repeat step 4 to add any other sequences of interest. In our example we will just add the EAEC genome Ec55989.

6. Now we need to specify the output file. Click the button marked '…' to select an output file. Navigate to the directory in which you want the output to appear. Now specify a name for the output file (e.g. 'mauve_output') and click 'Save'.

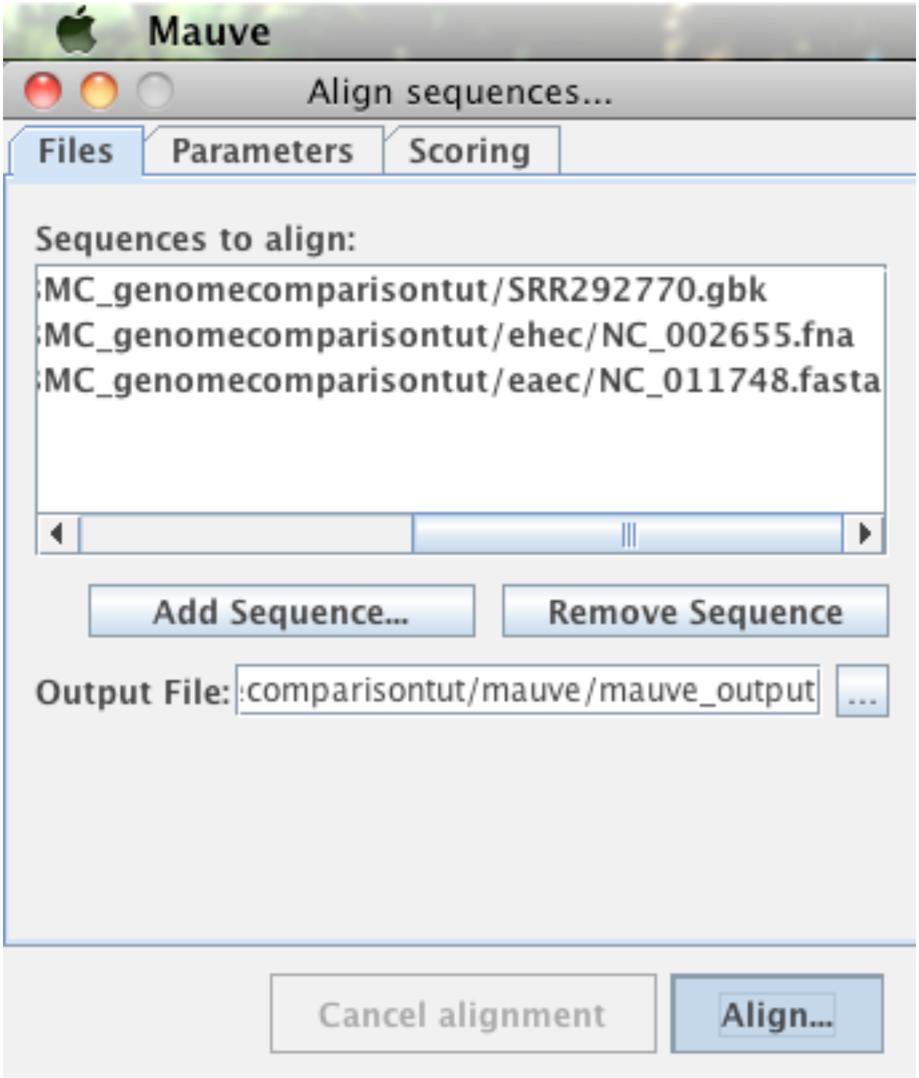

7. Click 'Align…' to run the alignment. This might take half an hour or so. A new window should appear marked 'Mauve Console' where the progress of the run will be displayed, including any error messages.

   If you encounter errors, check that you have specified the right files for input – they should all be fasta or multi-fasta sequence files, and can include up to one genome in Genbank format (to provide an annotation).

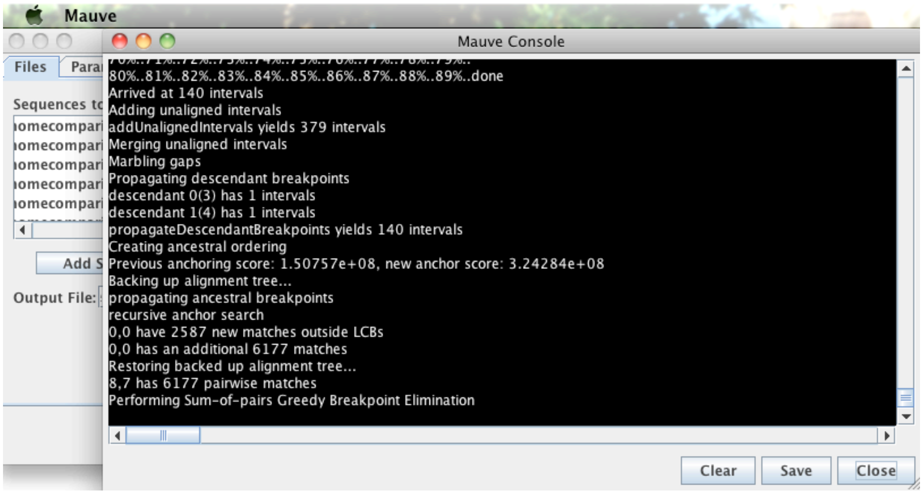

8. When the alignment is finished, the visualization tool will appear. To simplify the image a little, select View -> Style -> uncheck 'LCB connecting lines'. It should look like this:

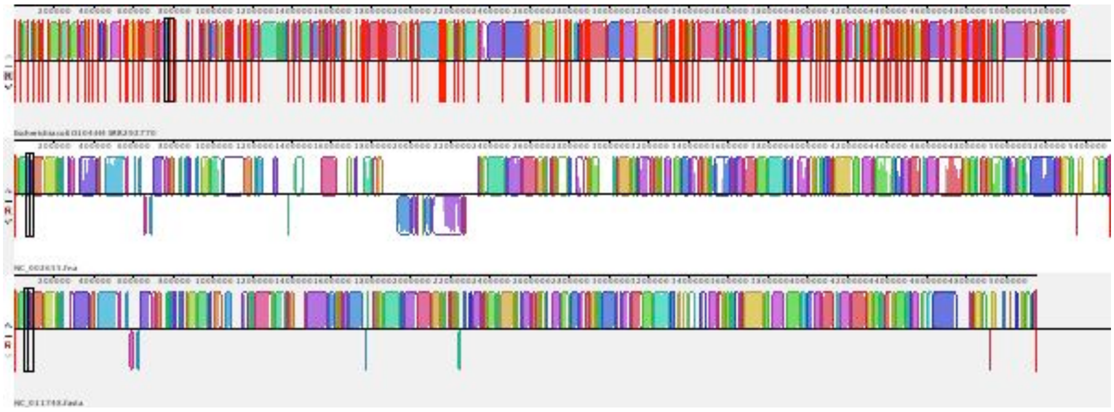

Row 1 = annotated O104 genome.
Row 2 = EHEC genome
Row 3 = EAEC genome
Coloured blocks indicate regions of sequence with homology in the other genomes.
Red lines indicate contig boundaries.

You can save a static image of what you are viewing by selecting Tools -> Export -> Export image…

9. Notice the EHEC genome has more 'white space', *i.e.* sequences not in homology blocks, meaning these sequences are missing from the new O104:H4 genome and Ec55989. The other genomes have fewer white blocks, as they share a lot of their genome sequence.

To see what the 'unique' sequences are in the O104:H4 assembly, zoom in by clicking the '+' magnifying glass at the top of the window until you see boxes appear under the O104 sequence; these are annotated genes.

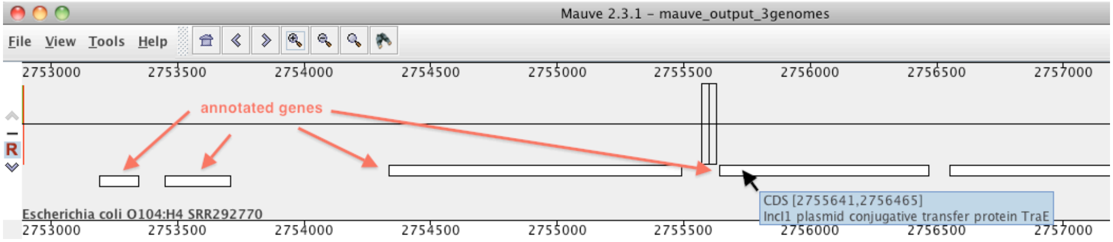

Scroll around to a region that is not within a coloured block, and mouse-over a gene to see its annotation. In our example, we are looking at a region of sequence in which IncI1 plasmid genes have been annotated. So, we know the O104:H4 genome assembly contains an IncI1 plasmid.

2.3 ACT – for detailed pairwise genome comparisons

Website: http://www.sanger.ac.uk/resources/software/act/ (To download, click the 'Downloads' tab) and look for the FTP download link for your operating system. Note that the download should contain Artemis as well as ACT.

Compatibility: Java based, available for Windows, Mac OS X, and Linux
This tutorial was created using ACT version 11.0.0 on Mac OS X.

Reference: Carver T. J,, Rutherford, K. M,, Berriman, M,, Rajandream, M. A., Barrell, B. G. and Parkhill J., "ACT: the Artemis Comparison Tool"**.** *Bioinformatics,* 2005. 21:3422-3. 10.1093/bioinformatics/bti553

Inputs: ACT can display pairwise comparisons between genomes. To do this it needs the genome sequences themselves (in fasta format or annotated sequence format such as Genbank or EMBL files) and a comparison file. Comparison files can be created on your computer if you have BLAST installed, or using an online tool like WebACT (http://www.webact.org/) or DoubleACT (http://www.hpa-bioinfotools.org.uk/pise/double_act.html), see steps 1-2 below.

Instructions

In this example we will visualize a comparison of our newly assembled and ordered *E. coli* O104:H4 TY-2482 contigs against enteroaggregative *E. coli* Ec55989 (accession NC_011748) and the EHEC genome EDL933 (accession NC_002655).

2.3.1 Generating comparison files for ACT

1. To generate the comparison file, you will need to have both of the genome sequences in single-fasta format. Generation of the comparison will not work with multi-fasta sequences such as those containing several contig sequences as output by Velvet or other assemblies. So, we first need to change the multi-fasta contig sequences file into a fasta file with a single entry, which includes all of our contig sequences concatenated together into one big sequence. An easy way to do this is to open the contig file in Artemis first.
    a. Launch Artemis
    b. Select File -> Open
    c. Navigate to the location of your contig file in fasta format and click 'Open'. The contig sequences should be displayed, with the boundaries of each contig marked up as a feature and coloured in alternative orange/brown colours.

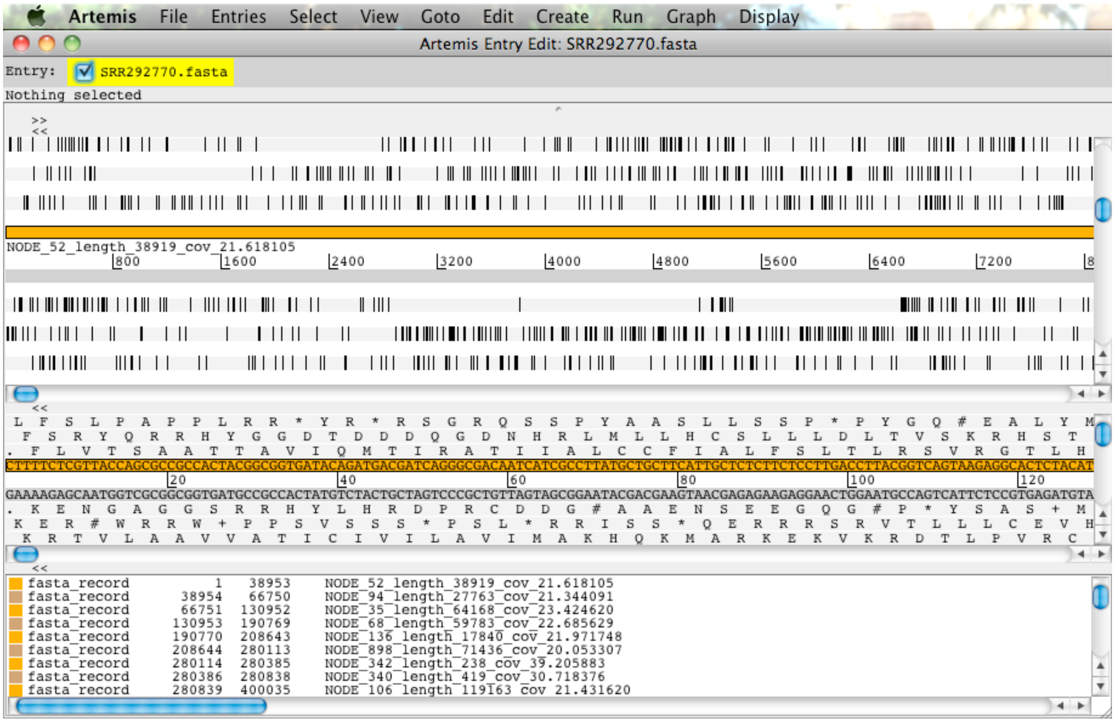

d. To write out the concatenated contig sequences to a single-entry fasta file, select File -> Write -> All Bases -> FASTA Format and save name the new file something like 'genomeXX_single.fasta' so you can easily identify this as a single-entry file.

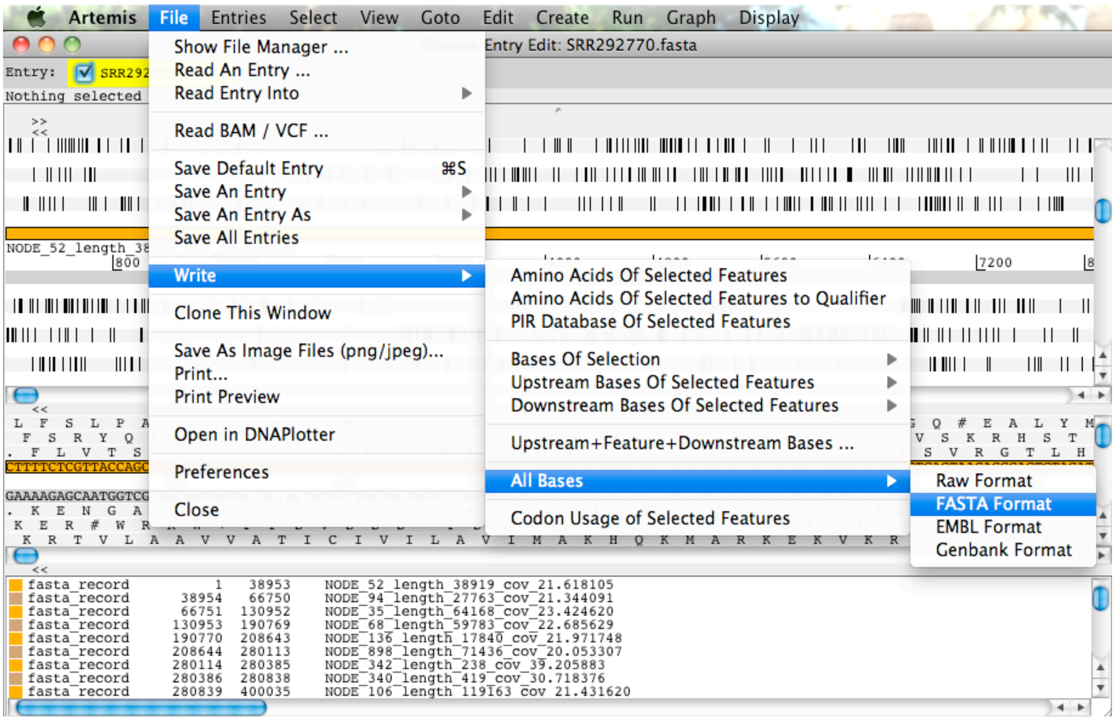

2. Generate a comparison between your single-entry fasta files by one of the following methods:

   a. If you have BLAST installed locally on your computer, open up a terminal and type:

      ```
      makeblastdb -in NC_011748.fasta -dbtype nucl

      blastn -query SRR292770_single.fasta -db
      NC_011748.fasta -evalue 1 -task megablast -outfmt 6
      > SRR292770_NC_011748.crunch
      ```

   b. If you prefer to use a web-based tool, go to the WebACT site (http://www.webact.org/) and click the 'Generate' tab at the top of the page. Under 'Sequence 1' paste in the accession for your reference genome, e.g. NC_011748. Under 'Sequence 2' click the 'Browse' button and navigate to your single-entry genome sequence file to compare. Click 'Submit'. It may take a while to upload your sequence (1-10 minutes), and a while longer for the results to be returned (1-60 minutes).

      When WebACT is finished, you will see a Results screen. Click 'Download files'. Enter a file name (a sensible choice is something that includes the full identifier of both genomes being compared, e.g. SRR292770_NC_011748.zip), download the file and unzip it. Inside will be a set of files including the input sequences; the comparison file is the one named ''. Rename it to something more informative (e.g. SRR292770_NC_011748.crunch) and copy it to the directory with your sequence files. (You can now delete the rest of the WebACT output.)

   c. You can also try the DoubleACT website (http://www.hpa-bioinfotools.org.uk/pise/double_act.html). Click the 'Browse…' buttons to upload your single-entry genome sequence file and the reference file for comparison, then click the 'Blastn' radio button, enter your email address and click 'Run genome blast'.

      When the comparison file is created, you will receive an email with a link to download the results. The comparison file is the one named 'genome_blast.result'. Right-click to save it to your computer in the same directory as your sequence files, and name it something more informative (e.g. SRR292770_NC_011748.crunch).

We generated comparison files for the *E. coli* O104 assembly vs Ec55989 (accession NC_011748), and for the *E. coli* O104 assembly vs EHEC genome EDL933 (accession NC_002655).

2.3.2 Viewing genome comparisons in ACT

1. Launch the ACT application

2. Select File -> Open

3. Initially, boxes for 2 sequence files and 1 comparison file will be displayed. Click 'more files…' to create boxes for a second comparison file and a third sequence file.

4. Click the 'Choose…' buttons to select each of your two sequence files and your comparison file. Note that you can load in your multi-fasta contigs file at this point for the *E. coli* O104:H4 genome. We want the *E. coli* O104:H4 assembly in the middle, with the comparisons to Ec55989 and EHEC above and below it, like this:

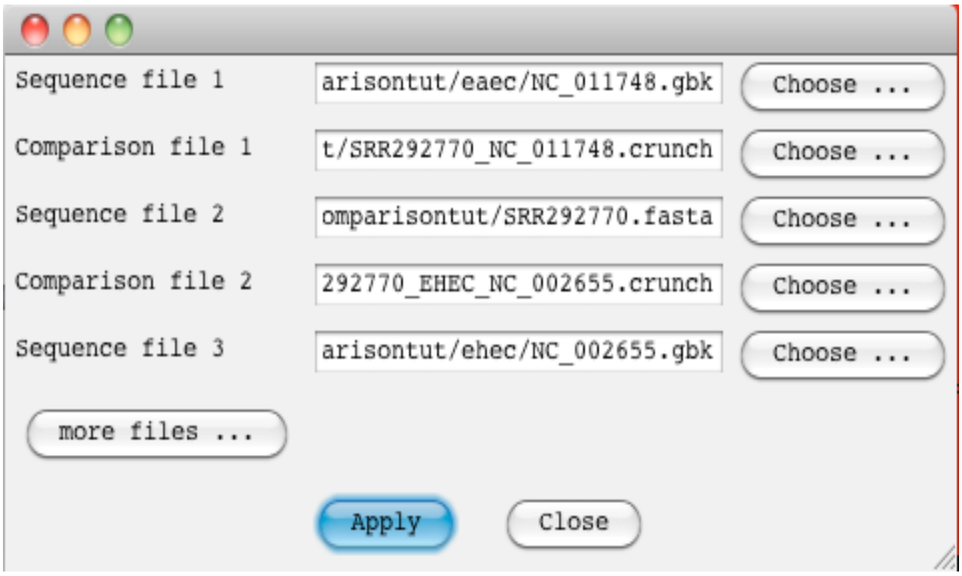

5. The comparison between the two genomes will be displayed. See the ACT manual for details of how to navigate around the viewer. Here, we are comparing the new *E. coli* O104:H4 genome assembly (bottom) with Ec55989 (top). We have zoomed out by clicking the down arrow at the bottom right of the window. Since our contigs were ordered against the Ec55989 genome, all the *E. coli* O104:H4 contigs with no homology to Ec55989 (i.e. no coloured bars linking them to Ec55989) appear at the end of the sequence.

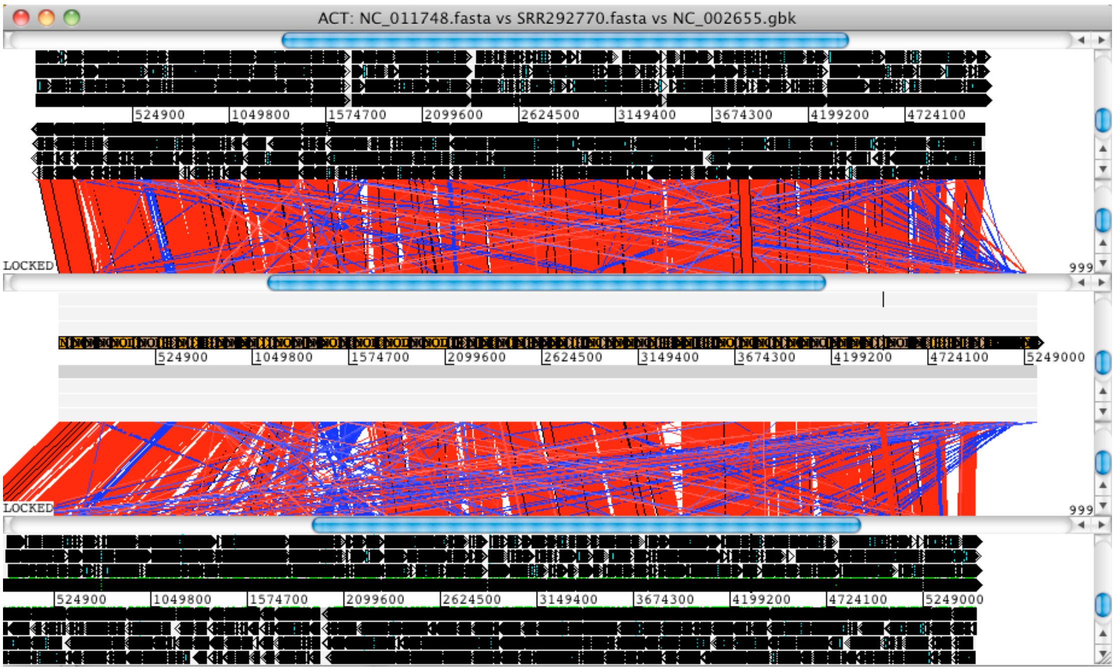

Zoom into this region by clicking on one of the unmapped contigs in this area and then clicking the up arrow to the side of the O104:H4 sequence.

2.4 BRIG – Visualizing reference-based comparisons of multiple sequences

Download from: http://brig.sourceforge.net/. The site contains download links, installation instructions, a manual and a tutorial which you may find useful.

Compatibility: Java based, available for Windows, Mac OS X, and Linux
This tutorial was created using BRIG version 0.95 on Mac OS X.

***Dependencies:** BRIG also requires BLAST be installed on your computer.
You can download BLAST+ from
ftp://ftp.ncbi.nlm.nih.gov/blast/executables/blast+/LATEST/. Ensure you select the file that matches your operating system, e.g. 'ncbi-blast-x.x.x+-universal-macosx.tar.gz' for Mac OS X or 'ncbi-blast-2.2.27+-win32.exe' for Windows.

Reference: Alikhan, N. F., Petty, N. K., Ben Zakour, N. L. and Beatson, S. A., "BLAST Ring Image Generator (BRIG): simple prokaryote genome comparisons", *BMC Genomics*, 2011. 12:402. PMID: 21824423

Instructions
NOTE: If you have not used BRIG before, you will probably find it useful to work through the BRIG tutorial available at http://brig.sourceforge.net/brig-tutorial-1-whole-genome-comparisons/ before working through the rest of our example.

1. Select your reference sequence and the location of your query sequences. In this analysis, we will use our de novo assembled *E. coli* O104-H4 contig sequences as the reference, and the EHEC and EPEC genomes as queries (see download links in 2.1). We will also include the sequences for the Stx2 phage and the LEE pathogenicity island.

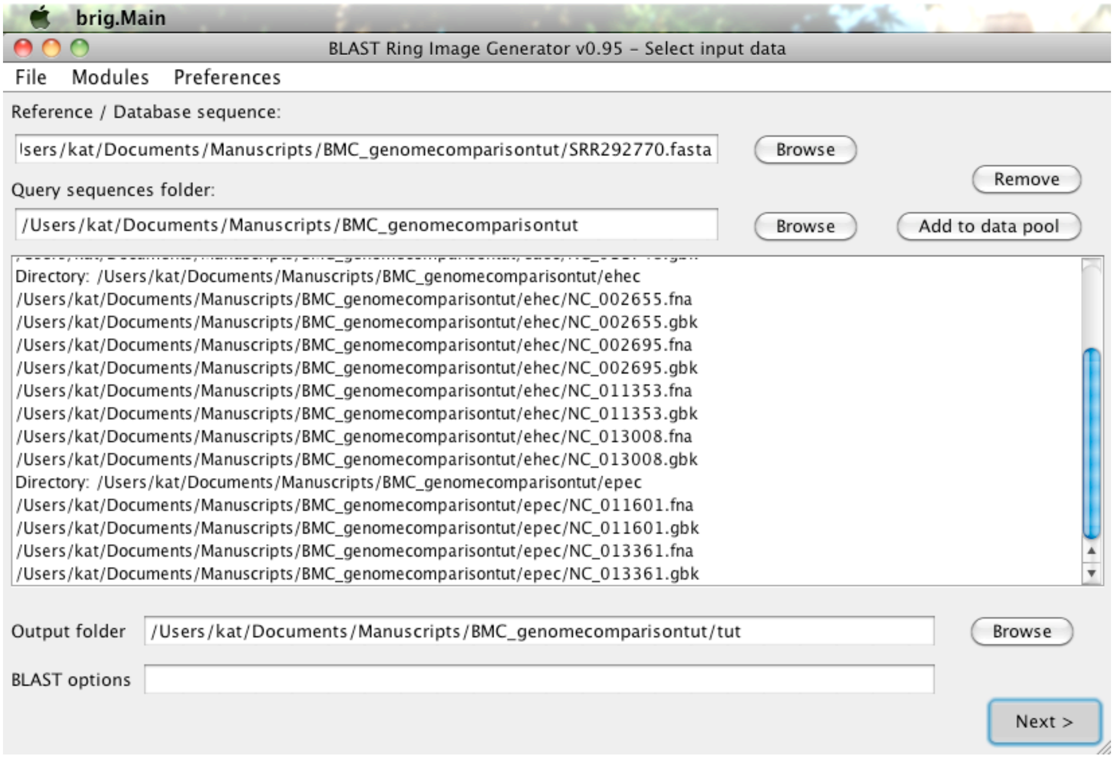

2. Click 'Next' to be taken to the 'Customize rings' window. This is where you can specify which query sequences you want to be represented by rings, and the order and colour they will be displayed.

3. Find the Ec55989 sequence in the 'data pool' box and click 'Add data'. Click the coloured box and change to colour to red. In the box marked 'Legend text:' type in a name for this ring, e.g. '1: Ec55989'.

4. Click 'Add new ring'. Now find the EHEC genome in the data pool and click 'Add data'. Set the colour to purple and change the legend text to '2: EHEC str 1'.

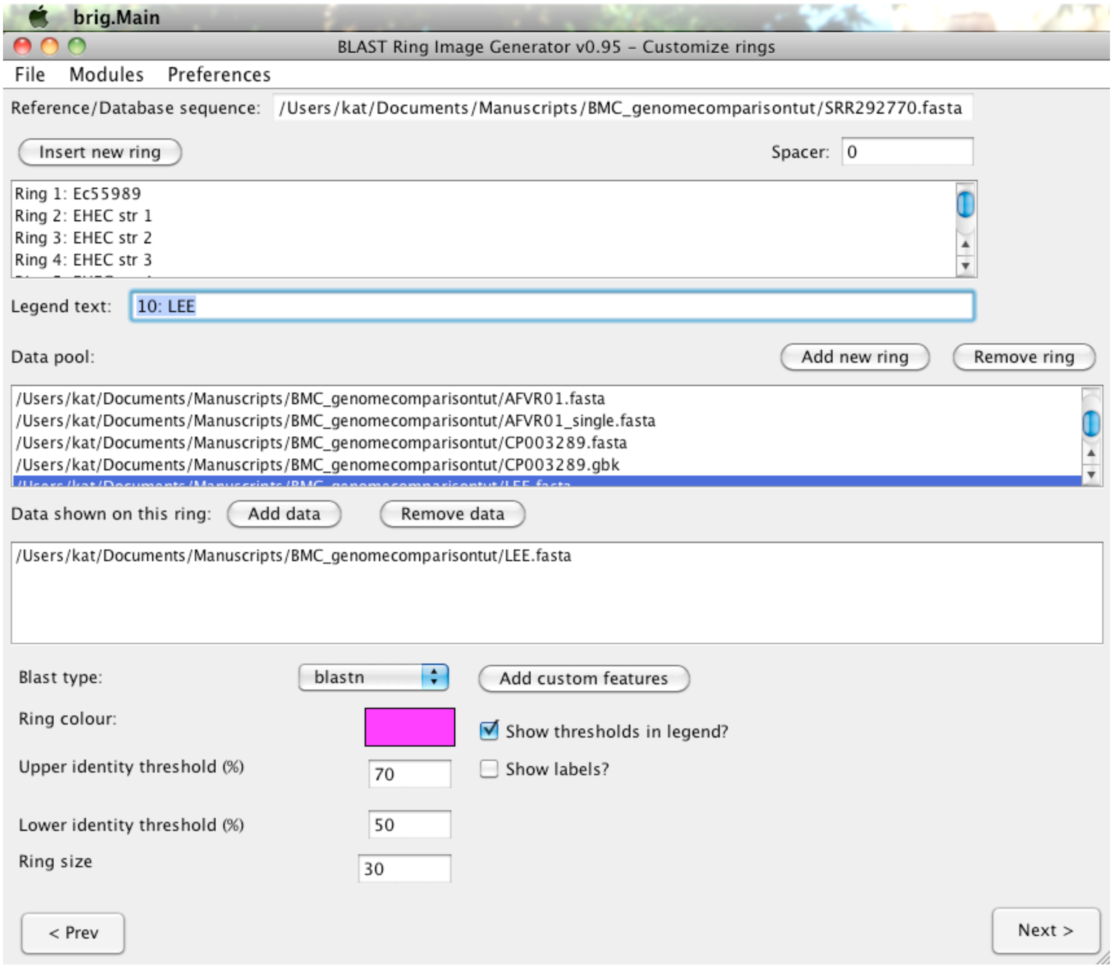

5. Repeat step 5 with the remaining EHEC and EPEC genomes. We used the 4 EHEC genomes listed in this tutorial under 'Downloads' and coloured them all purple, two EPEC genomes coloured blue, and one atypical EPEC genome coloured green.

6. Go to the Preferences menu and select Image Options. Under the 'Global settings' tab change the 'Width' field to 2500. This will make the image canvas wide enough to display the legend text next to the ring image, without obscuring the image itself. Click 'Save & close'.

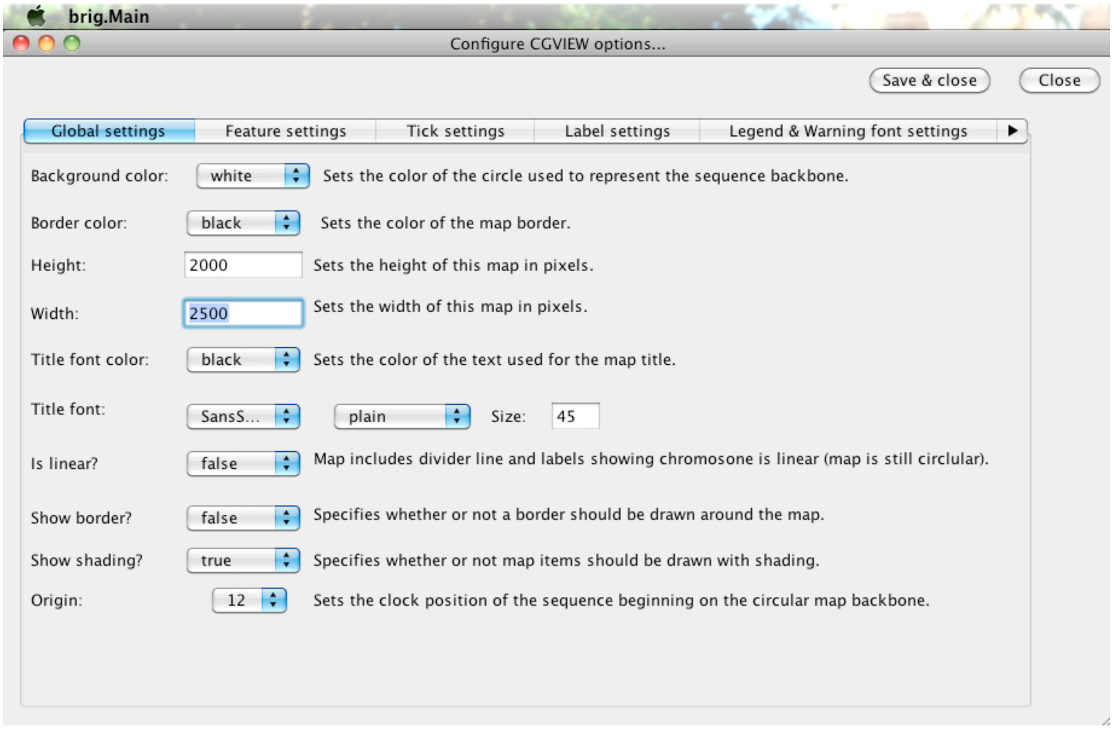

7. Click 'Next' and enter a title for your image (this will be printed in the middle of the circular diagram). Click 'Browse' and navigate to where you want the output to be saved, then type in a name for the output file (this will be a single image file) and select the format for the image (e.g. png). Click 'Submit'.

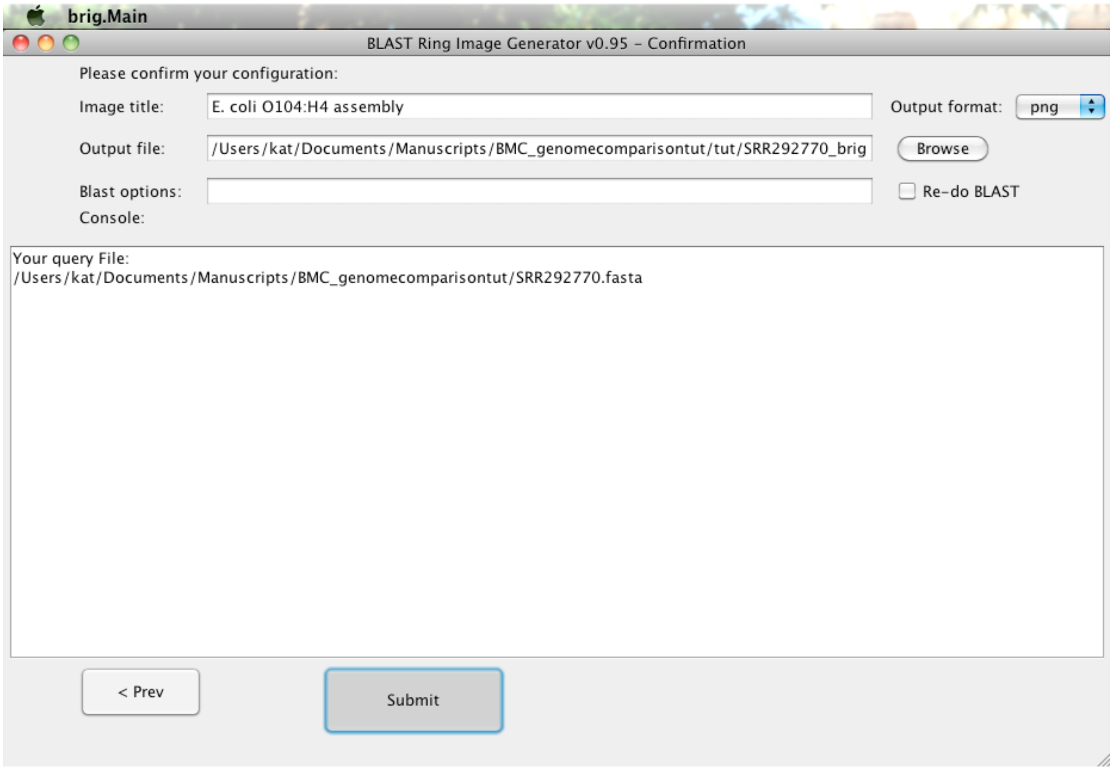

8. While BRIG is running, it will print details of its progress on the console within the same window where you just pressed 'Submit'. When it tells you it has finished, go to where you asked for the output to be saved and open the image file to view the result.

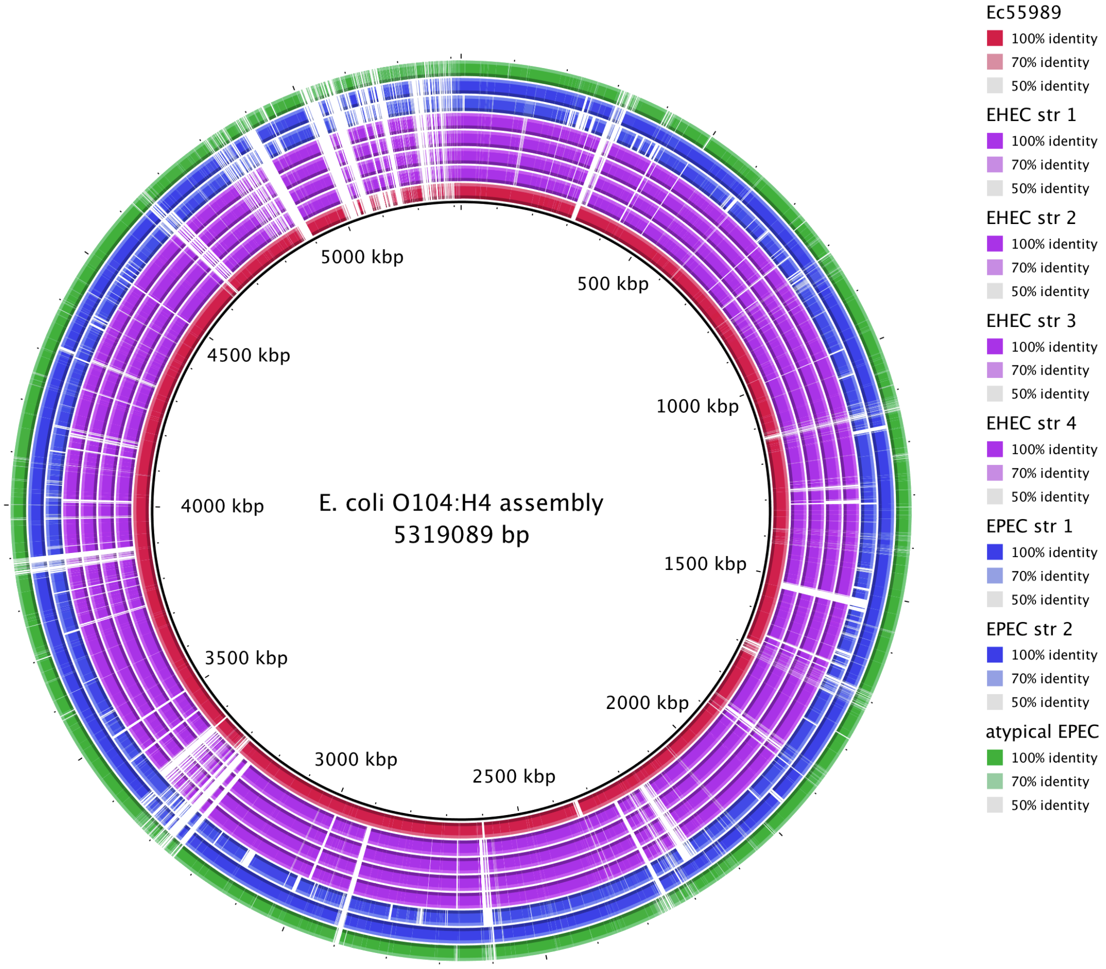

It is easy to see that, in terms of gene content, the novel O104:H4 outbreak strain is closest to EAEC strain Ec55989 (red), then the atypical EPEC strain E110019 (green). There are several regions of the outbreak strain's sequence that are missing from the EHEC and EPEC strains.

9. An alternative way to make the comparison is to use an EHEC genome as the reference sequence, to see how much of the characteristic EHEC sequences are present in the outbreak genome. Click the 'Prev' button to get to the 'Customize rings' window, then click 'Prev' again to get to the input data window. Change the reference sequence to EHEC strain EDL933 and click 'Next'. Now change ring 2 to be the new O104:H4 genome... Click on 'Ring 2' in the list of rings in the first box; click the old EHEC strain in the 'data' box and click 'Remove data'; find the O104:H4 file in the data pool and click 'Add data'. Change the Legend text to '2: O104:H4' and change the ring colour to black. We also added the Stx2 phage (orange) and the LEE pathogenicity island (purple), to make it easy to see where these regions are. Click 'Next' and change the image title and output filename to indicate that the reference sequence is an EHEC strain, then click 'Submit' to generate the image.

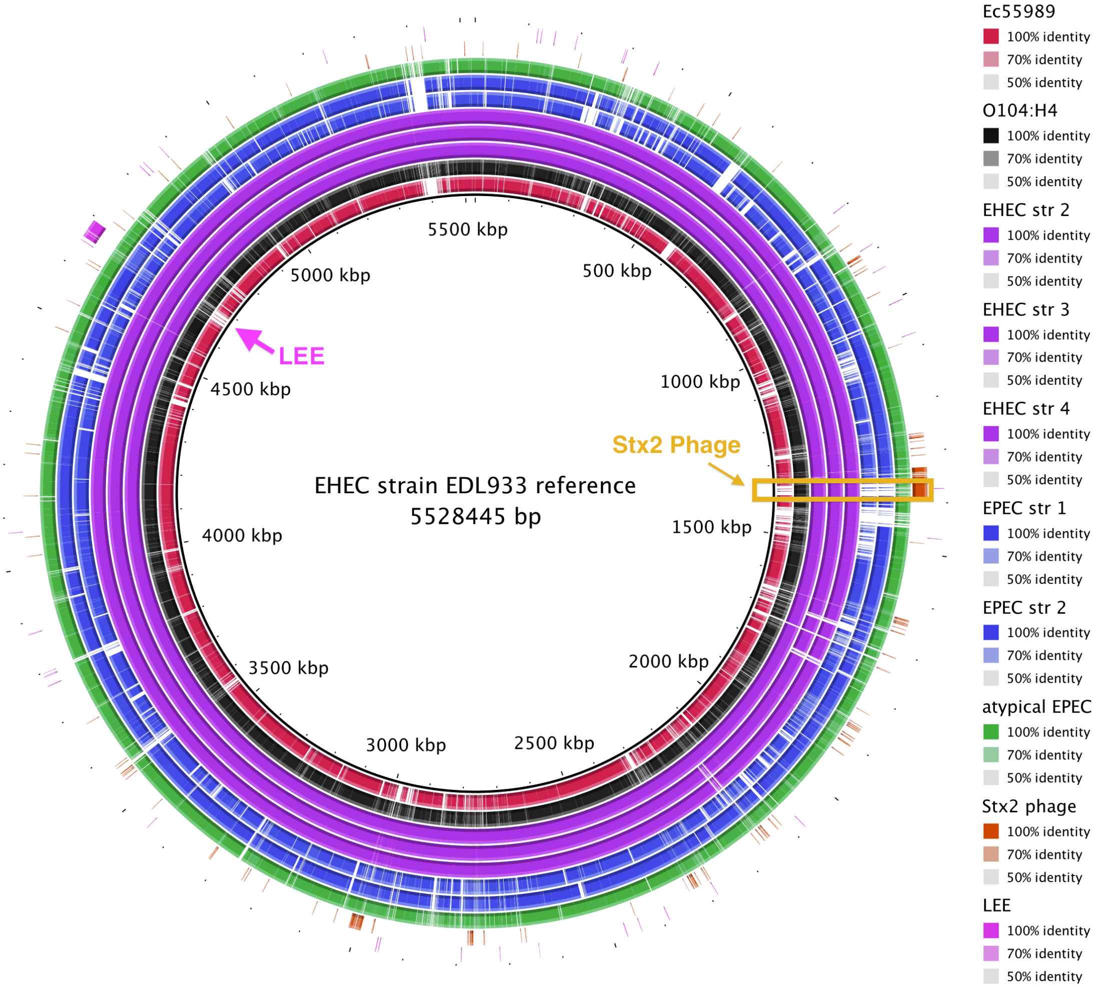

10. Try changing the reference strain to the Ec55989, EPEC or atypical EPEC genomes and see how the figures and the interpretations change.

3. Typing and specialist tools

3.1 PHAST – for identification of phage sequences

Type: Web service
URL: http://phast.wishartlab.com/
Reference: Zhou, Y., *et al.*, "PHAST: a fast phage search tool"*. Nucleic Acids Research*, 2011. 10.1093/nar/gkr485.

Input: Contigs in FASTA format (single or multiple fasta)

Outputs:
- Summary Table (summarising the location of prophage sequences)
- Detailed Table (giving the locations of individual genes within the prophage)
- Circular genome map (showing the locations of prophages within the genome)
- Linear maps of each prophage (showing the individual genes)

See the PHAST website for more detailed documentation.

3.2 ResFinder – for identification of resistance gene sequences

Type: Web service
URL: http://www.cbs.dtu.dk/services/ResFinder/
Reference: Zankari, E., *et al.*, "Identification of acquired antimicrobial resistance genes*.*" *J Antimicrobial Chemother*, 2012. 10.1093/jac/dks261.

Input: Contigs in FASTA format or reads (will assemble first).

Outputs: List of resistance genes identified within the sequences

3.3 Multilocus sequence typing

Type: Web service
URL: http://cge.cbs.dtu.dk/services/MLST/
Reference: Larsen, M. V., *et al.*, Multilocus Sequence Typing of Total Genome Sequenced Bacteria*. J Clin Microbiol*, 2012. 10.1128/JCM.06094-11.

Input: Contigs in FASTA format or reads (will assemble first).
Parameters: Select the MLST database to query.
Outputs: Top hitting alleles for each locus used in the MLST scheme, and the sequence type (ST) assigned to that combination of alleles.

3.4 PATRIC – online genome comparison tool

For an introduction to what PATRIC can do, try looking at their analysis of the *E. coli* O104 genome, posted at http://enews.patricbrc.org/1172/e-coli-outbreak-new-comprehensive-comparisons/
